# Spatial recognition and semi-quantification of epigenetic events in pancreatic cancer subtypes with multiplexed molecular imaging and machine learning

**DOI:** 10.1038/s41598-025-90087-z

**Published:** 2025-02-22

**Authors:** Krzysztof Szymoński, Natalia Janiszewska, Kamila Sofińska, Katarzyna Skirlińska-Nosek, Dawid Lupa, Michał Czaja, Marta Urbańska, Katarzyna Jurkowska, Kamila Konik, Marta Olszewska, Dariusz Adamek, Kamil Awsiuk, Ewelina Lipiec

**Affiliations:** 1 Department of Pathomorphology, Jagiellonian University Medical College, Grzegórzecka 16, Cracow, 33-332 Poland; 2 Diagnostyka Consilio Sp. z o.o, Cracow, Poland; 3 M. Smoluchowski Institute of Physics, Jagiellonian University, Cracow, Poland; 4 Doctoral School of Exact and Natural Sciences, Jagiellonian University, Cracow, Poland; 5 Department of Pathomorphology, University Hospital in Cracow, Cracow, Poland; 6 Department of Pediatrics, Jagiellonian University Medical College, Cracow, Poland

**Keywords:** Spatial epigenomics, Epigenetic modifications, DNA conformation, Z-DNA, Pancreatic cancer, Molecular imaging, Cancer epigenetics, DNA methylation, Acetylation, Methylation, Machine learning, Molecular conformation, Imaging studies, Raman spectroscopy

## Abstract

Genomic alterations are the driving force behind pancreatic cancer (PC) tumorigenesis, but they do not fully account for its diverse phenotypes. Investigating the epigenetic landscapes of PC offers a more comprehensive understanding and could identify targeted therapies that enhance patient survival. In this study, we have developed a new promising methodology of spatial epigenomics that integrates multiplexed molecular imaging with convolutional neural networks. Then, we used it to map epigenetic modification levels in the six most prevalent PC subtypes. We analyzed and semi-quantified the resulting molecular data, revealing significant variability in their epigenomes. DNA and histone modifications, specifically methylation and acetylation, were investigated. Using the same technique, we examined DNA conformational changes to further elucidate the transcriptional regulatory mechanisms involved in PC differentiation. Our results revealed that the foamy-gland and squamous-differentiated subtypes exhibited significantly increased global levels of epigenetic modifications and elevated Z-DNA ratios. Overall, our findings may suggest a potentially reduced efficacy of therapeutics targeting epigenetic regulators for these subtypes. Conversely, the conventional ductal PC subtype has emerged as a promising candidate for treatment with epigenetic modulators.

**Supplementary Information:**

The online version contains supplementary material available at 10.1038/s41598-025-90087-z.

## Introduction

The wide range of pancreatic cancer (PC) phenotypes causes difficulties in diagnosing, treating, and determining patient prognosis due to ambiguous, non-subtype-specific results. PC shows a variety of phenotypes regarding its histology (morphology), genetic alterations, and epigenetic modifications (EMs). The subtyping appreciates different aspects of the PC picture; consequently, molecular (transcriptomic), clinical, or histological subtypes were distinguished^1^. Studies have revealed differences in their biology, resulting in different prognoses and treatment efficiencies^1–7^.

Although it might be difficult to correlate and link different systems of PC subtyping, some attempts have been made^1,8^. Nevertheless, important practical aspects of PC include its histological subtypes. The routine pathological practice, which establishes the details of PC diagnosis used in clinical management, exploits histopathological subtyping. This is partially caused by the cost-effectiveness of routine diagnostics, which currently does not allow for extended molecular testing of each patient. Antibody-based assays for histone and DNA modifications are being developed^9^; however, caution must be taken when introducing new techniques, and proper standardized procedures should be implemented before^10^. The transcriptomic subtypes distinguished by Collison et al.^2^, Moffit et al.^3^, and Bailey et al.^4^ are highly exploited in PC research^1,11^. Currently, however, they are not included in pathological diagnosis or clinical patient management procedures. Recognizing these subtypes is largely based on expensive RNA profiling, even though other approaches have been proposed^11^. Moreover, only a few molecular subtypes were distinguished, whereas morphological and biological PC presentations are much more common (over thirteen WHO and four non-WHO subtypes^5,12^). Experience learned from the classification of central nervous system tumors based solely on the profile of DNA methylation taught us the potential of EMs in practical tumor subtyping^5,13^. A multitude of these tumors were recognized and new entities were revealed that had not been identified before^13^.

PC emerges in a well-documented fashion, originating as pancreatic acinar cells through acinar-to-ductal metaplasia or as pancreatic duct epithelial cells. The initial process of carcinogenesis, driven by genetic alterations, is followed by subsequent modifications (i.e., mutations in KRAS, TP53, CDKN2A, or SMAD4), depicted as dysplastic changes in flat lesions called pancreatic intraepithelial neoplasia (PanIN) or cystic lesions, such as the most prevailing intraductal papillary mucinous neoplasm (IPMN)^5^. However, genetics alone does not sufficiently reflect a variety of PC subtypes^14^.

The emerging role of epigenetic alterations in PC has been recognized. Recently, we first linked some of the most common PC histological subtypes with variabilities in DNA methylation^15^. Moreover, recent studies on targeted therapy approaches have focused on this particular aspect of cancer progression^16^. Indeed, epigenetic alterations are excellent targets for PC therapeutics because they are reversible^16^. Notably, the change in the PC epigenome is very dynamic, not only in highly proliferating cancer cells but also as a reaction to the tumor environment and its host, showing a *modus operandi* that even justifies a travesty of a “mafia” within the “society of the body”^17^. Tumor-stroma crosstalk, another highly exploited subject of PC research, impacts the tumor epigenome^18^. This drives cancer spreading (meaning the ability to invade and metastasize), a logical consequence of the theory of evolution and its natural selection mechanisms^19^.

Recent advances in cancer research have revealed three main dimensions of the transcriptomic machinery. First, the nucleotide sequence of the genome is prone to rearrangements, duplications, deletions, and other alterations^20^. In the past, it was thought that the DNA sequence solely determines basic transcription into proteins. However, at least two other underlying mechanisms alter the amino acid sequences in the resulting proteinogram. The local DNA three-dimensional (DNA conformation) structure directly affects the binding of different proteins involved in the DNA transcription process^21,22^. Finally, DNA and histone modifiers affect chromatin modeling, thus blocking or favoring the transcription of certain DNA sequences^23^.

DNA appears in different secondary structures, known as conformations. The most prevalent is the right-handed double helix B-DNA, which builds up the DNA backbone^24^. Other double helix conformations (non-B) include right-handed A-DNA and left-handed Z-DNA. Additional DNA structures include DNA bubbles, cruciform, three-stranded H-DNA, four-stranded G-quadruplexes, and others^24,25^. In cancer research, Z-DNA has been exploited in the context of new therapeutic approaches^26^. Recently, the Z-form of DNA was shown to be involved in the ability of cancer cells to suppress apoptosis via the immune response, an escape mechanism of cancer cells from so-called immune checkpoint blockade (ICB) therapeutics^27^. Proteins containing Z-DNA-specific domains (called the Zα domain), such as adenosine deaminase acting on RNA 1 (ADAR1) or Z-DNA binding protein 1 (ZBP1)^28–31^, selectively bind to the Z-DNA form. Activation of ZBP1 leads to autoinflammation and necroptotic cell death, although it is negatively regulated by ADAR1^32–34^. In cancer cells, ADAR1 is used to induce immune silencing and prevent termination, thus bypassing the therapeutic effect of ICB^28^. Recognition of the Z-DNA distribution in PCs might provide insight into cancer-induced ADAR1 activation.

EMs are posttranslational processes that are crucially important in PC biology. The constant interplay between EMs leads to variable gene expression, which is dynamically regulated as a reaction to the environment or cellular requirements (i.e., highly proliferating cancer cells)^16^. EMs include DNA and histone modifications. DNA methylation (DNA-m) most commonly occurs in areas of cytosine-guanine dinucleotides (so-called CpG islands) and controls the transcription of specific genes, increasing or blocking it. On the other hand, histone modifications locally alter the state of chromatin, causing chromatin to transform into dense heterochromatin or loose euchromatin. The latter allows DNA transcription and gene expression. EMs are driven by enzymes that add or remove methyl or acetyl groups to CpG islands or histone amino acids. These proteins are grouped into so-called *“writers”*, which include DNA methyltransferases, histone lysine methyltransferases, and histone acetyltransferases. Another group called “*erasers”* contains DNA demethylases, histone lysine deacetylases (HDACs), histone lysine demethylases (KDMs), and protein arginine methyltransferases. Consequently, histone acetylation leads to transcription activation, whereas histone methylation depends on the site of the residue and the degree of modification^16^.

Blocking some of the enzymes responsible for EMs in PC (such as specific HDACs or KDMs) are considered potential targets currently in clinical trials. This emerging role of epigenetic profiling in cancer therapy was excellently reviewed in^14^ and^35^. Another possible therapeutic approach involves blocking ADAR1 or activating ZBP1 to support the effects of ICBs and re-enable cancer cell apoptosis via the immune response^26,28^. Nevertheless, to successfully utilize these drugs in PC therapy, researchers must properly address PC heterogeneity and subtyping because of suspected variable responses. Indeed, most trials of PC therapy to date have yielded unsatisfactory results^35^.

Here, we aimed to spatially recognize EMs and DNA conformations in PC subtypes. To do that, we employed an innovative approach relying on Raman hyperspectral mapping (RHM) combined with advanced machine learning techniques, including unsupervised autoencoders (AEs) and convolutional neural networks (CNNs). RHM is a method of unlabeled molecular imaging based on Raman spectroscopy that allows for the study of cancer tissues with submicrometric resolution, visualizing cellular components up to even individual chromosomes^36,37^. Adjacent pixels of spectral measurements are merged to form a so-called hyperspectral image. The information collected with RHM contains molecular interactions in the studied sample in an all-in-one manner, which authorizes the conclusion of the sample molecular structure and, thus, the contents of DNA or proteins, such as histones, and their secondary structures. Nevertheless, the need for high-quality RHM measurements and complex analysis of the resulting data, until recently, prevented the use of RHM in high-class cancer tissue exploration. Only a few reports are available describing DNA-m using Raman and infrared spectroscopy techniques^38,39^, and some attempts have been made to detect histone acetylation^40,41^. However, these studies primarily examined isolated DNA strands or individual cells rather than complex tissue samples. Recently, our group reported on DNA-m patterns among several PC histological subtypes obtained with RHM^15^.

In this study, PC tissues of six histologically different subtypes were compared to benign control pancreatic ductal (CTRL) tissues. Specifically, we evaluated conventional ductal adenocarcinoma (cPDAC), adenocarcinoma derived from intraductal papillary mucinous neoplasm (IPMC), predominantly foamy-glands carcinoma (FG), predominantly large duct type carcinoma (PLD), and squamous differentiated adenocarcinoma (SD). These are the most common subtypes of ductal adenocarcinoma, which is the most common form of PC^12^. Furthermore, for comparison, we added the ampulla of Vater adenocarcinoma (AVAC), which is a cancer entity not classified as a form of PC according to the WHO; nevertheless, it develops in the main duct of the pancreatic opening into the duodenum (the ampulla of Vater). The diagnosis of AVAC vs. PC is frequently histopathologically challenging because of similarities in their morphologies and immunoreactivity^15^. Thus, practically, both PC and AVAC are considered when diagnosing a patient with a pancreatic tumor.

Above all, we spatially show and semi-quantify the heterogeneity of EM in PC. In particular, we recognized and analyzed the global distribution of DNA-m, methylated lysine (Lys-m), acetylated lysine (Lys-a), and methylated arginine (Arg-m). Additionally, we complete these findings with the results of Z-DNA and B-DNA conformation distributions. Consequently, we identified the FG and SD PC subtypes as potentially less vulnerable to epigenetic regulator-targeting therapies.

## Results

### Artificial neural networks precisely identify cancer cell nuclei

After including patients and selecting relevant tissue sections, followed by marking areas of interest (cancer and benign tissues), we obtained high-quality RHM maps of PC tissue samples. All-in-one hyperspectral data requires exact spectral classification to allow further analyses and interpretation. This process is of key significance for separating relevant information from other parts of tissue samples, such as tumor stroma, inflammation infiltrates, necrosis, benign tissues, or even cancer cell cytoplasm (in the context of exclusively studying cancerous nuclei). For a broader discussion on the importance of such specificity, we refer the reader to our previous papers^36^.

**Fig. 1:**
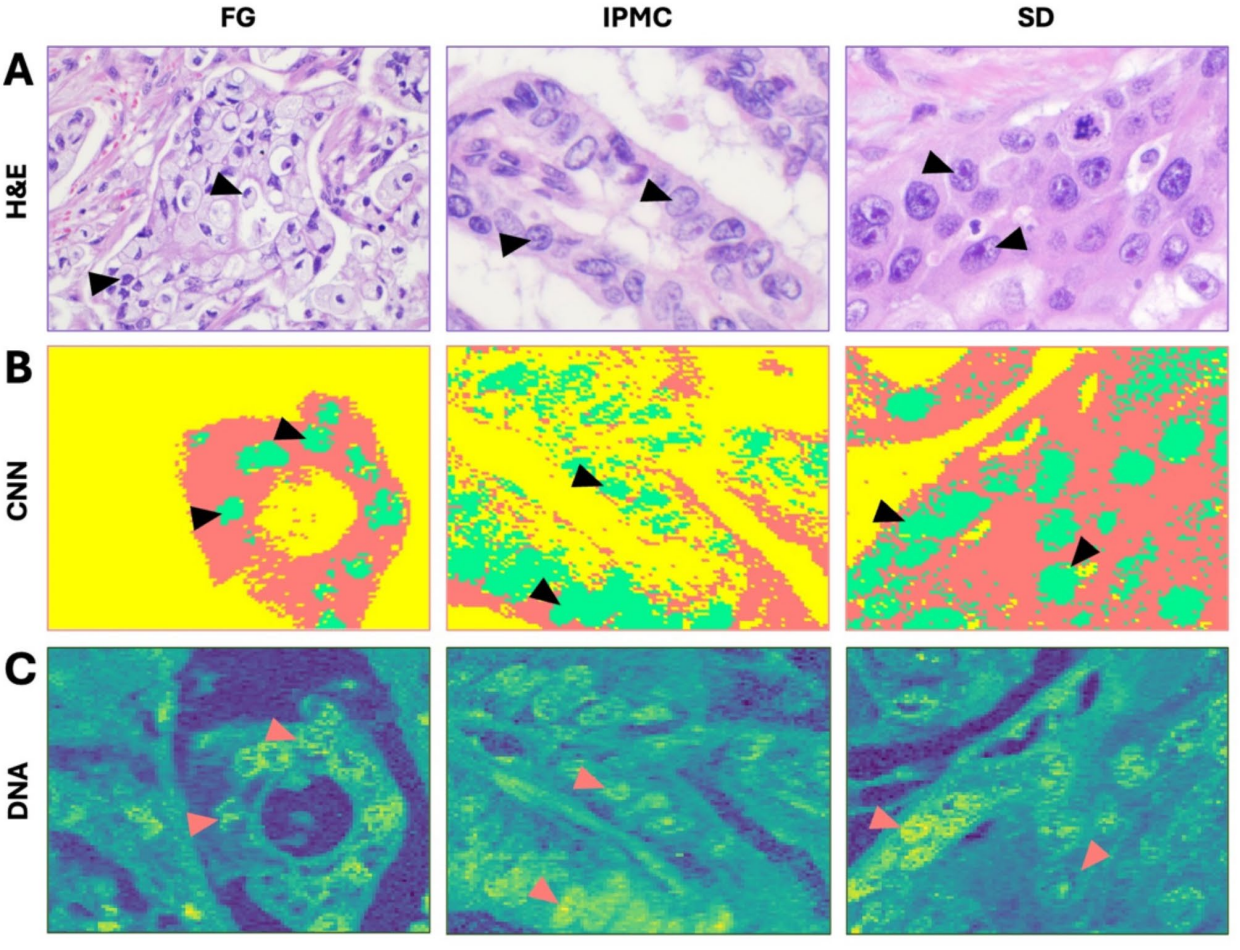
Neural network-based classification of pancreatic cancer nuclei and histological slides of corresponding areas. Each section represents a hematoxylin-and-eosin (**A** - H&E) image of the PC area, the corresponding image generated by the CNN classification (**B** - CNN) into areas of cancer cell nuclei (green), cytoplasm (red), and other tissues (yellow), and a DNA correlation map (**C** – DNA). Arrowheads indicate cancer cell nuclei. The resemblance of H&E and DNA regarding subcellular component discrimination is noticeable (i.e., nuclear shape, size, crowding, or nucleoli details). Note that H&E staining is performed on a subsequent slide from the tissue block. *(H&E*,* original magnification 400×)*

For the task of spectral data classification, the most efficient and accurate methods are CNNs^36^. Here, we used CNNs to classify RHM spectra into cancerous nuclei, cytoplasm, and tumor stroma. The process of spectral preprocessing using AE and CNN training is described in the *Methods* section of the manuscript. The accuracy of successful classification (so-called validation accuracy) of the CNN training reached between 96 and 100%, and the results of that classification were plotted as CNN-prediction maps (Fig. 1). The subcellular components, such as the nuclei, are well-projected in CNN representations (Fig. 1B) based on hematoxylin & eosin-stained slides (Fig. 1A). Further sections of the manuscript describe the presented DNA-correlated map images (Fig. 1C), which reflect PC cells in more detail.

### Molecular imaging with reference DNA correlation reveals highly detailed subcellular structures of pancreatic cancer and benign control tissues

Once prepared and classified, the RHM spectra could become the subject of proper analyses to reach the goals of our study. Here, we aimed to reveal the spatial distribution of EMs in PC subtypes. We searched for a way to extract specific information about DNA and histone modifications from the general data accessible in RHM spectra. We hypothesized that each spectrum of the RHM map could be correlated with a reference spectrum of certain EMs, resulting in a correlation score. We chose to study the most prevalent EMs in PC; consequently, Raman spectra of isolated DNA, lysine (Lys), and arginine (Arg) were measured and juxtaposed with measurements of methylated DNA, methylated Lys, methylated Arg, and acetylated Lys. Supplementary Figures S1A – S4A show the reference Raman spectra of these isolated DNA and amino acids, which were used for further analyses and comparisons.

For the spectral correlation scoring, we chose the Pearson correlation coefficient (Pearson R), as it provided the best results in complex samples such as PC tissues (see the *Discussion* for details of this choice). The relative Pearson R correlation maps obtained by comparison of the reference DNA spectra revealed the ability to reflect the subcellular components of PC tissues with astonishing accuracy and were comparable to those of hematoxylin and eosin-stained slides (Fig. 1A vs C).

**Fig. 2:**
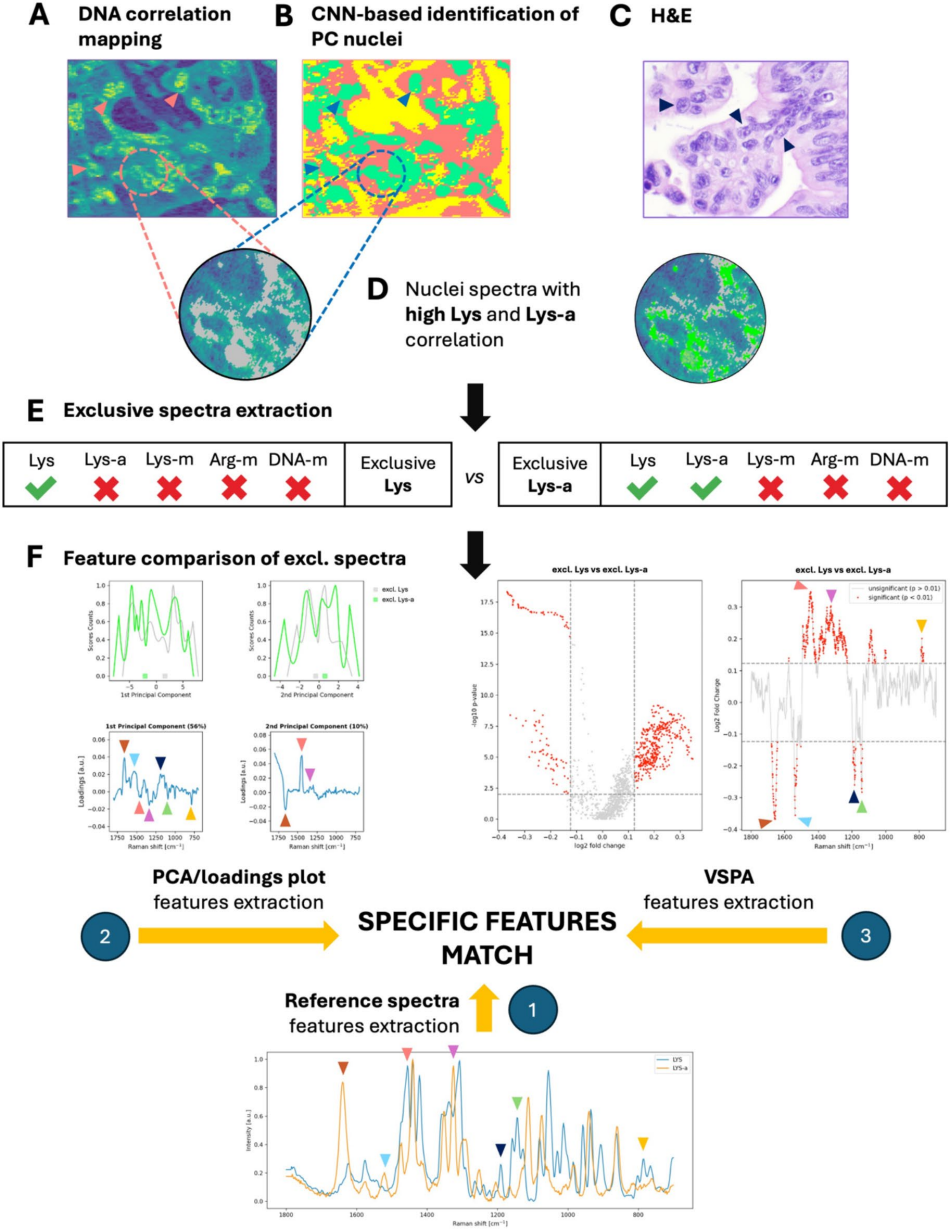
Validation of the spectral correlation scoring approach workflow (based on Lys-a). The correlation score was validated threefold. First, a correlation score-based heatmap with the reference DNA spectrum was generated, revealing a highly detailed view of subcellular components, including the nuclei (**A**). This step confirmed the spatial distribution of the DNA, which was excellently depicted with the corresponding hematoxylin-and-eosin (H&E) slide (**C**). Note that H&E staining is performed on a subsequent slide from the tissue block; thus, some areas are not precisely the same, although a clear resemblance of tumor morphology is observed (i.e., cellular nuclei size, shape, crowding, and chromatin condensation). Arrowheads indicate cancer cell nuclei. Further steps involved CNN classification into areas of cancer cell nuclei (**B**, green, arrowheads), cytoplasm (**B**, red), and other tissues (**B**, yellow), which allowed the selective extraction of nuclear spectra. From nuclei areas revealed by the CNN, we identified those that were “exclusive” for each epigenetic modification (**D**). Thus, for each modification (DNA-m, Lys-m, Lys-a, and Arg-m), a correlation score with a reference spectrum was measured, and a binary status of *positively* (1) or *negatively* (0) modified was applied, depending on that score (a positive 3rd quartile of correlation score distribution was used as the cutoff value). Consequently, the *exclusive spectra* for Lys-a comprised only those that were Lys-positive and Lys-a-positive but were negative for all other modifications. In circles (**D**), a high-resolution heatmap of a nucleus with superimposed exclusive spectra of Lys (**D**, round map, gray) and Lys-a (**D**, round map, green) is presented, further highlighting the spatial distribution of nuclear DNA and histones (Lys). In the next step (**E**), we extracted *exclusive spectra* for Lys and Lys-a for subsequent spectral feature comparison (**F**). The goal at this point was to identify characteristic features that differentiated our reference spectra of Lys and Lys-a (**F**, 1). These were then identified in the so-called loadings plot of principal component analysis (PCA) of *exclusive* Lys and *exclusive* Lys-a spectra (**F**, 2) and confirmed in another method of analysis based on a volcano plot, which combines the statistical significance of differentiating spectral features with a significant fold change in that differentiation (volcano plot-based spectral peak analysis – VSPA), revealing the most notable features (**F**, 3). The color arrowheads in (**F**) show the corresponding features characteristic of Lys-a identified in the reference spectra, PCA, and VSPA. A further description of our approach is presented in the *Methods* section. *(C*,* H&E*,* original magnification 400x; F-1*,* reference Lys and Lys-a spectra comparison; F-2*,* PCA score counts plot*,* loading plot; F-3*,* VSPA volcano plot*,* significant peaks plot)*

The visual representation of the relative Pearson R between tissue samples and the reference DNA spectrum served as a proof-of-concept, proving that the method is suitable for spectral data comparison even in complex tissue samples. However, to extend this hypothesis in the context of EM imaging, which cannot be visually confirmed, as in the case of DNA, further validation was needed. We approached this threefold, as schematically presented in Fig. 2 and described in detail in the *Methods* section. Briefly, in addition to visual DNA confirmation, we identified differentiating spectral features characteristic of each of the EMs and confirmed them by (i) reference spectra matching, (ii) principal component analysis (PCA), and (iii) so-called volcano plot-based spectral peak analysis (VSPA) (see Fig. 2nd legend for the explanation).

### High-resolution multiplexed imaging of pancreatic cancer nuclei reveals variability in epigenetic modification levels

**Fig. 3:**
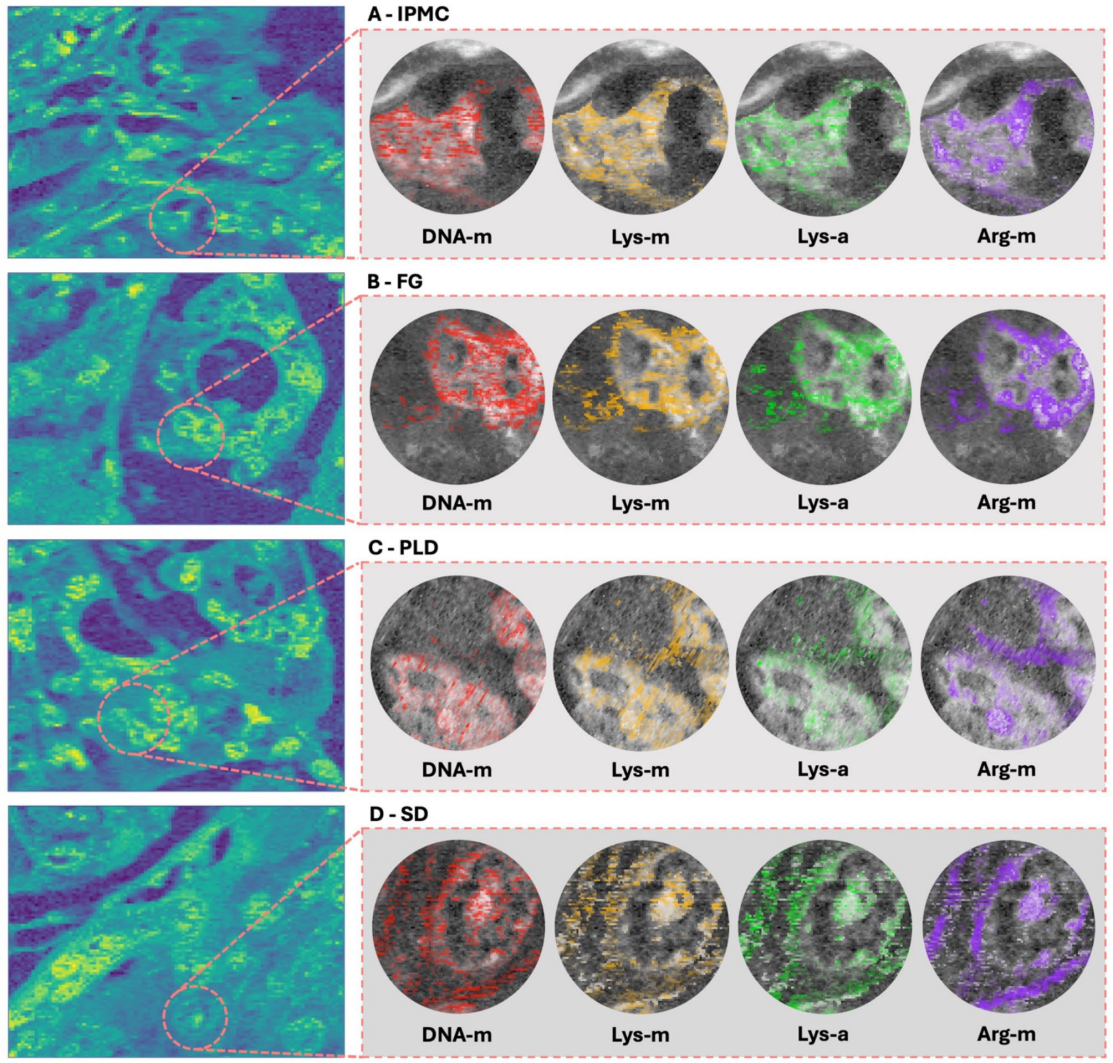
Multiplexed high-resolution insight into the heterogeneity of pancreatic cancer nuclei. High-power views of the PC nuclei with superimposed overlays of EMs show variability in their spatial distribution. In the zoomed-in views of selected nuclei from the IPMC (**A**), FG (**B**), PLD (**C**), and SD (**D**), each color dot represents the EM from transparent to oblique according to the level of that modification. The EM levels are determined in areas that are DNA, Lys, or Arg-positive for DNA-m, Lys-m/Lys-a, and Arg-m, respectively. The whole slide views (left side of the figure) and the background images in the highlighted EM views reflect a relative DNA correlation heatmap with a reference DNA spectrum.

Mapping based on relative correlation provided excellent large-scale images of PC tissues. After the identification of cancerous nuclei by the CNN classification, we aimed to capture the heterogeneity of nuclear morphology and EM occurrence. By choosing specific regions of the previously RHM-mapped tissues, we conducted subsequent molecular imaging at the highest resolution authorized by the diffraction limit (a measured pixel size of 250 nm, resulting in an actual resolution of approximately 500 nm given the Nyquist criterion). These new data were similarly correlated with the reference DNA spectrum, resulting in high-quality images of single-nucleus areas. The next step was to superimpose the highlighted areas of EMs and show multiplexed images that depict the variability of PC nuclei in terms of morphology and EM distributions. Figure 3 shows an example of the multiplexing of selected PC subtype nuclei.

### Semi-quantified expression of epigenetic modifications significantly differs between pancreatic cancer subtypes and benign pancreatic duct tissues

**Fig. 4:**
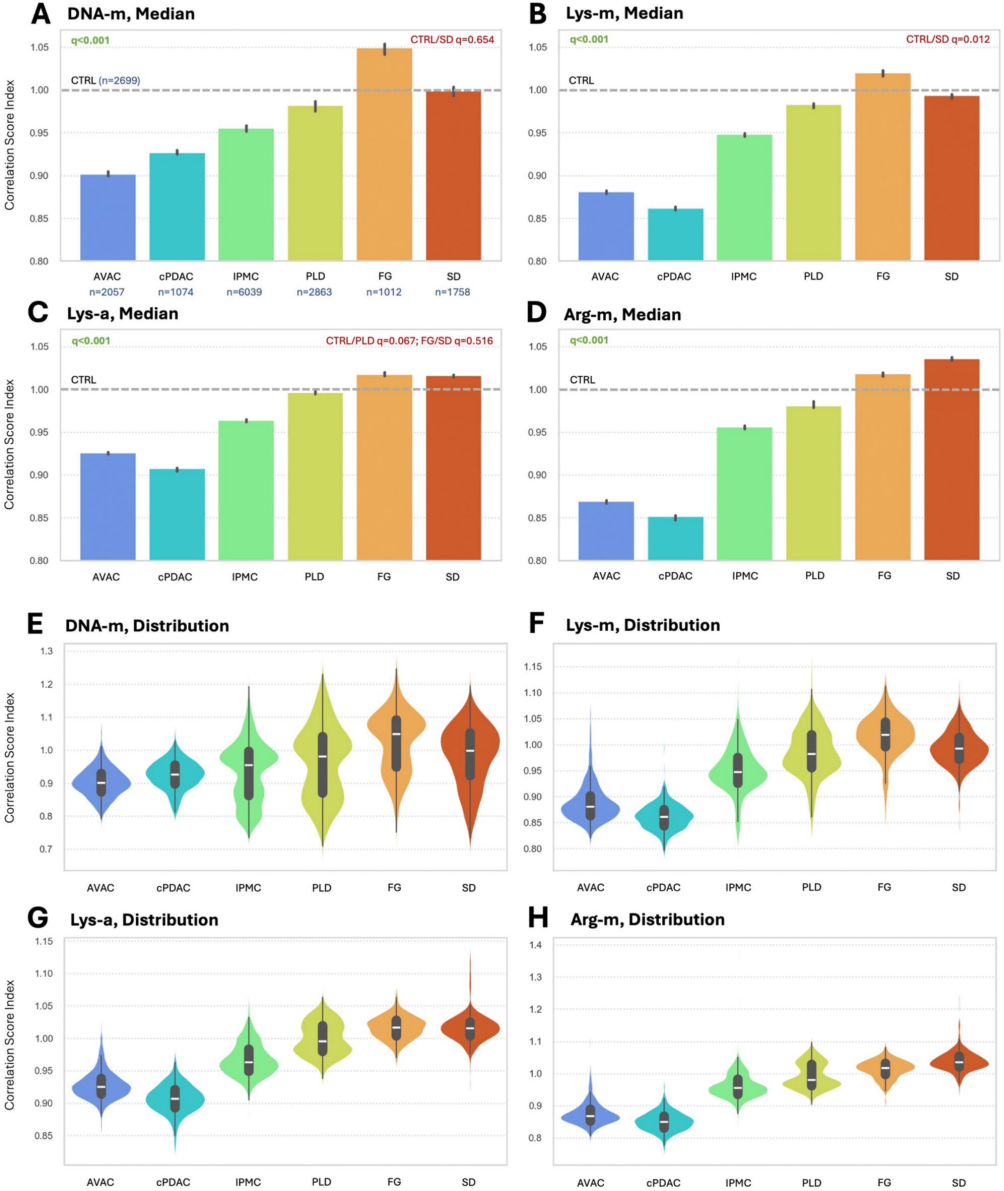
Variable expression of DNA and histone epigenetic modifications among pancreatic cancer histological subtypes. The median levels (**A**-**D**) and distributions (**E**-**H**) of correlation-based EMs in nuclei identified by the CNN in all studied PC samples are presented as a ratio to the CTRL. The gray dashed lines at 1.0 show the CTRL level of a particular EMs. The AVAC and cPDAC PC subtypes were the most distinct from the CTRL at all EM levels, with AVAC presenting slightly less DNA-m than cPDAC, and oppositely at other modifications. The levels of EMs increase at IPMC and PLD, with the highest for the FG and SD. A particularly surprising finding is the hypermethylation (compared to CTRL) of DNA and Lys in FG and Arg in SD. *(q*,* adjusted p-value of Dunn’s post hoc for the Kruskal‒Wallis test; n*,* number of spectra correlated)*

Although the visualization of EM heterogeneity in PC subtypes revealed intriguing results, the semi-quantification of these relationships is of greater value in terms of unequivocal pathological interpretation and discussion. All spectra from the areas classified as nuclei by the CNN and positive for DNA, Lys, or Arg were correlated with reference EM spectra and analyzed. In Fig. 4A and D, the relative differences among PC subtypes and benign control pancreatic ductal tissues are presented for each of the EMs. It is important to note that the CTRL group spectra were extracted precisely from the pancreatic ductal tissues, omitting all other components, such as acini, pancreatic islets, or inflammation infiltrates, which could falsify the results. Almost all comparisons show significant differences, with only individual cases not statistically meaningful. Specifically, in DNA-m, no distinction is shown for CTRL vs. SD. In the Lys-m comparisons, only the CTRL vs. SD were similar, whereas in the Lys-a comparisons, the separations of the CTRL vs. PLD and FG vs. SD were not statistically meaningful. All groups revealed significant differences in the Arg-m correlation. Figure 4E and H present the distribution of these findings among the studied samples.

### The ratio of left-handed Z-conformation of DNA varies among pancreatic cancer subtypes

**Fig. 5:**
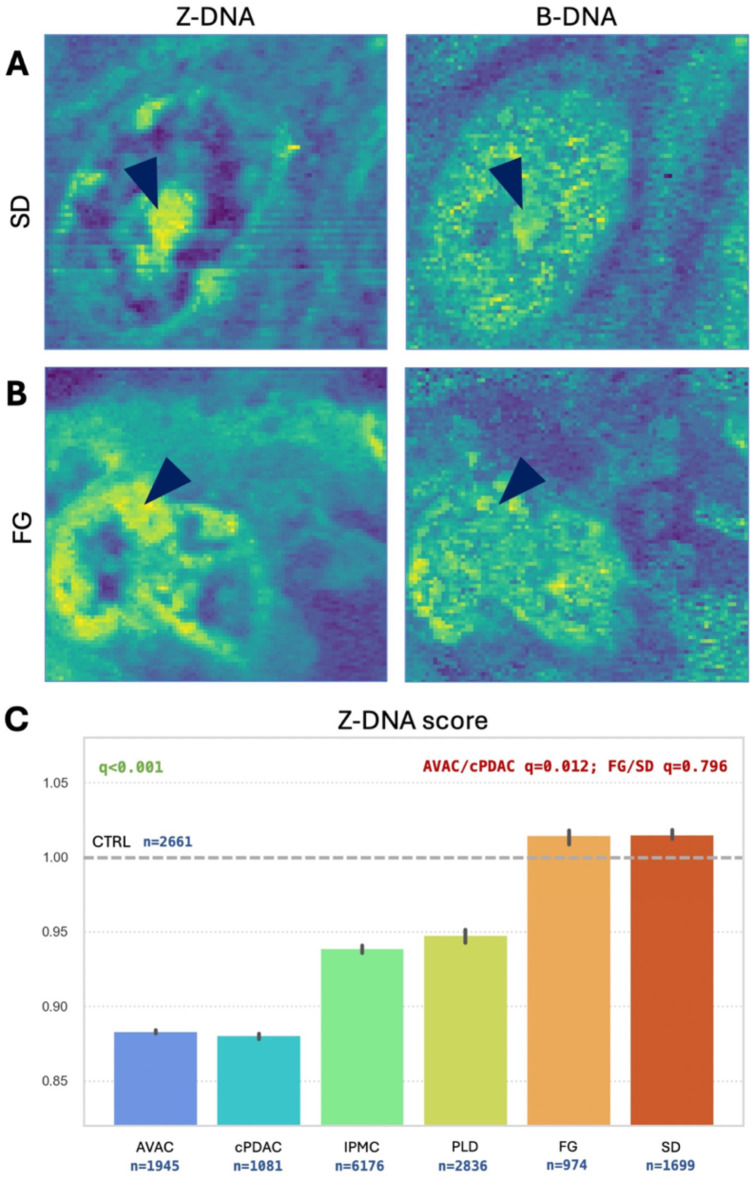
Distribution of left- and right-handed DNA conformations in pancreatic cancer nuclei. The distributions of the Z-DNA and B-DNA conformations are shown for selected PC subtypes, specifically the SD (**A**) and FG (**B**) subtypes. A more intense color correlates with a higher level of a specific conformation. B-DNA is the dominant conformation, whereas Z-DNA seems to favor the nucleoli and peripheral areas of the nuclei. Arrowheads mark the nucleoli. Semiquantitative analysis revealed significant differences among some PC subtypes, with FG and SD having the least amount of B-DNA and the most amount of Z-DNA, whereas cPDAC and AVAC presented opposite results. The Z-DNA score plot (**C**) was correlated with that of the CTRL samples (dashed gray line at 1.00). The score is the ratio of the Z-DNA expression level divided by the B-DNA expression level. *(q*,* adjusted p-value of Dunn’s post hoc for the Kruskal‒Wallis test; n*,* number of spectra correlated)*

To visualize and semi-quantify the distributions of DNA conformations, we utilized the same technique, which proved successful in the case of EMs. First, we measured the Raman spectra of isolated reference DNA in different conformations. The resulting reference spectra are shown in Supplementary Figure S6. Then, we looked for the same features in the RHM maps of the PC samples by correlating each of the map pixel spectra with the reference measurements. The score of that relative correlation was plotted, showing local variabilities in Z-DNA and B-DNA distributions (Fig. 5A and B). This visualization confirmed the predominant, more diffuse B-DNA localization in PC nuclei. On the other hand, Z-DNA is located in the nucleolus and peripheries of the nucleus. Next, we semi-quantified the relationships between DNA conformations in all studied groups of PC. The Z-DNA score, which is the ratio of Z-DNA relative to B-DNA, is presented in Fig. 5C. In that figure, PC subtypes show a grouping of AVAC with cPDAC, IPMC with PLD, and FG with SD. Additionally, the Z-DNA content of the last group was similar to that of the CTRL samples. Overall, it is interesting that lower EM levels are associated with lower Z-DNA levels. Again, the FG and SD groups clustered together with the CTRL group, as shown by the EM distribution results (Fig. 4).

### General analysis of spectral features confirms the distinct biology of some of the pancreatic cancer subtypes

**Fig. 6:**
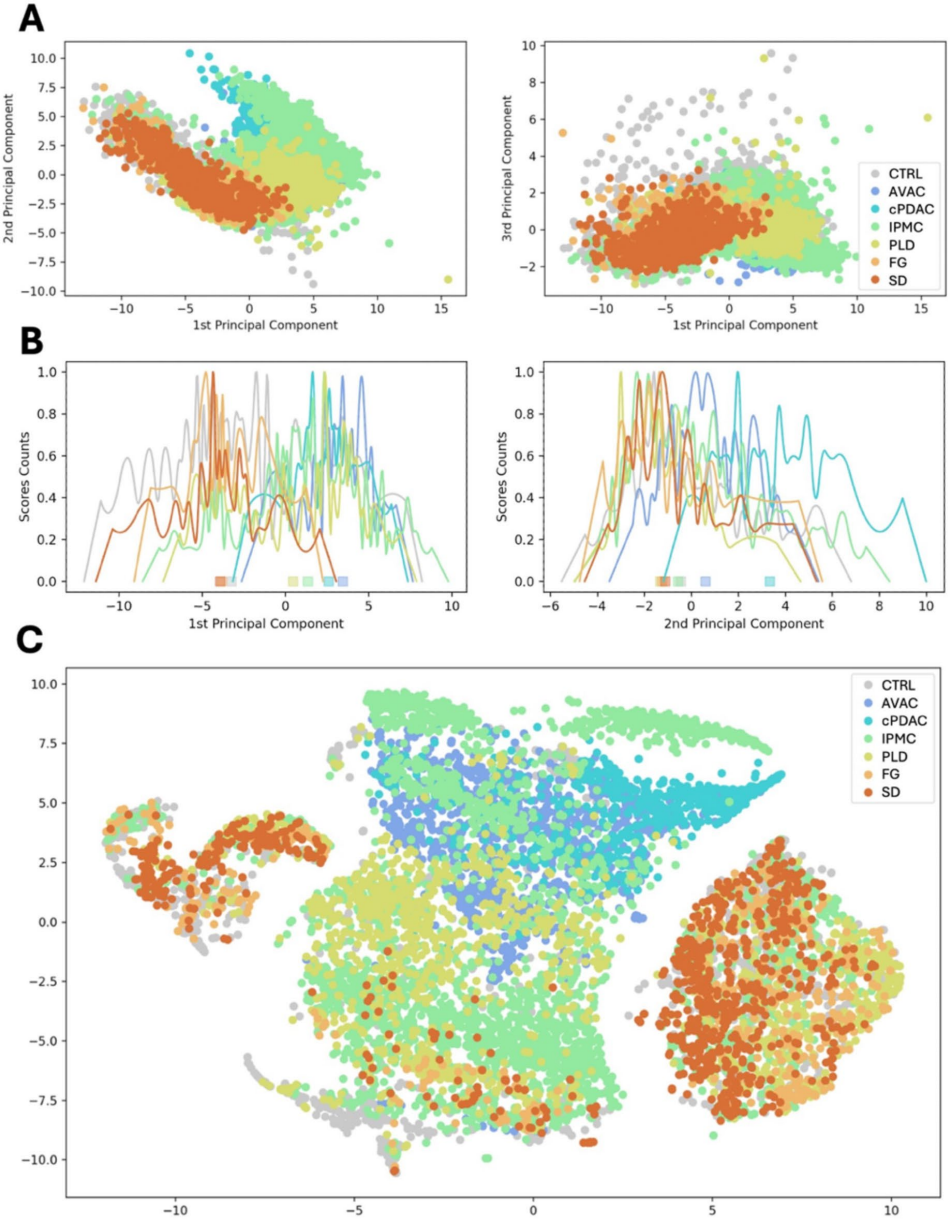
The grouping of pancreatic cancer subtypes based on their molecular interactions. The spectra obtained from DNA-positive areas of all studied histological subtypes of PC were separated by principal component analysis (PCA) and t-distributed stochastic neighbor embedding (t-SNE). PCA scores plot (**A**) and score counts plot (**B**) allocate FG, SD, and CTRL to one extreme, similar to t-SNE, which also groups these types. On the other extreme of PCA are sequentially placed PLD, IPMC, cPDAC, and AVAC. In (**B**), the squares at the bottom mark the median values of scores along principal components for each PC group, depicting their clustering. The t-SNE PLD groups were separate from the IPMC, cPDAC, and AVAC groups; however, the t-SNE PLD group participated in one branch of the first group (FG and SD), showing the common features of FG, SD, and PLD. Both PCA and t-SNE highlighted significant common features of FG with SD and cPDAC with AVAC; however, both groups were located on opposite sides. Interestingly, this analysis, although performed on all nuclear spectra, showed the same tendency as the results of the semiquantification of EMs or DNA conformation (Figs. 4 and 5). Further interpretation of this separation is shown in Supplementary Figure S7.

The recognized pattern of distinct EM and DNA conformation levels in FG, SD, and less for PLD from those of AVAC, cPDAC, or IPMC presented in Figs. 4 and 5 was confirmed with a general spectral feature comparison. We used two methods widely used for feature-based grouping of multidimensional data, namely, PCA^15,36^ and t-distributed stochastic neighbor embedding (t-SNE)^13^. In both methods, a clear separation between PC groups is observed, as depicted and briefly described in Fig. 6. Further analysis and interpretation of the differences in spectral features are presented in Supplementary Figure S7.

## Discussion

The importance of tumor subtyping in pathological diagnosis is well-known^42^. Specific subtypes define the biology of tumors and determine clinical outcomes. Along with tumor grading, histological subtypes are assigned to diagnosed lesions to categorize neoplasms into classes of predictable behavior. The malignancy potential, thus its ability to spread through metastases, nodal or perineural involvement, and patterns of that spread, are described through histological subtyping^7,12^. Moreover, even though the information obtained with proper subtype assignment is substantial, it is the most convenient and cost-effective way for both the diagnostic pathologist and the clinical oncologist who recommends further management of the patient. The treatment of the disease is determined by its histological subtype and grade^12,43^. Moreover, the genetic testing to identify molecular targets follows the histological pre-diagnosis. Naturally, the recognition of epigenetic mechanisms in cancer biology should involve the same convenience. Here, we revealed the variable EMs and DNA conformations in PC histological subtypes and identified those that significantly differed from the others.

In our study, we utilized a well-known correlation approach (the Pearson R), although it has not yet been applied for studying complex tissues. Along with other correlation-based techniques, it has proven efficient in Raman spectra matching^44,45^. Some authors have suggested that other methods of spectral matching are more accurate than Pearson R^46^. Thus, the Euclidean distance, cosine similarity, or squared first-difference cosine similarity were some of the proposed methods^46^. Nevertheless, after evaluating all of the mentioned techniques, we chose Pearson R as the best fit for spectral comparison in complex tissue samples (more details are provided in the *Methods* section). Notably, some aspects should be taken into account when considering the correlation of spectral data between isolated DNA or isolated amino acids and the correlation of spectra from complex PC tissues. It is to be expected that the latter should contain much more cumulative molecular data, at the same time joining intensities of multiple molecules and interfering with each other. Therefore, one should not expect the direct correlation to be high but rather pay attention to the relative relations, that is, the contrast between adjacent pixels in the correlation-plotted map image.

In the context of PC tumorigenesis and progression, DNA-m was confirmed to play a crucial role. There are two distinct epigenetic trends regarding DNA-m in tumors, including PC. First, the methylation of cytosine in CpG islands of individual gene promoters causes the silencing of tumor suppressor genes^47,48^. The second mechanism involves global cytosine demethylation (hypomethylation). PC is globally hypo-DNA-methylated^15,49,50^, which results in chromosomal instability (CIN), rapid gene mutations, and cancer progression^51,52^. CIN is considered a driver of cancer metastasis and chemoresistance^53^.

Furthermore, DNA-m interacts with other chromatin modifiers, such as HDACs or KDMs, to increase tumor suppressor gene silencing. It was established that increased expression of some HDACs in PC (i.e., HDAC1, HDAC2, HDAC3, HDAC4, or HDAC7) leads to deacetylation at Lys residues in PC histones^54–57^. Similarly, increased activity of some KDMs (i.e., KDM1A or KDM3A) causes histone Lys demethylation^58,59^. Moreover, the levels of histone trimethylation at Lys 27 (H3K27me3) were found to be generally decreased in PC^60^, whereas the total loss of H3K27me3 was associated with increased aggressive behavior of the tumor^60^. These findings were observed despite the overexpression of the histone-lysine methyltransferase EZH2 (enhancer of zeste homolog 2), which is the enzymatic driver of H3K27me3^50,61^. Reports on histone methylation at Arg (a specific locus or global expression) in PC are very sparse^62^.

Here, we report global hypomethylation of DNA and histones (at both Lys and Arg residues) and histone hypoacetylation (at Lys residues) in PC tissues compared to benign controls (Fig. 4). Most importantly, our results show that among its subtypes, PC is significantly heterogeneous in terms of EM expression. The presence of hypo-DNA-m in cPDAC, AVAC, and IPMC was recently confirmed by our group^15^. However, that work revealed only results limited to the methylation status of DNA, leaving the most important histone modifiers and the Z-DNA levels unrecognized. Moreover, the most important PC subtypes (i.e., FG, SD, and PLD), which we currently present, have not been investigated. In the present study, these subtypes exhibited EM patterns distinctly different from the current knowledge on PC (i.e., that PC is globally hypo-DNA-m). Specifically, the FG subtype was slightly hypermodified in all EM comparisons (q < 0.001, Dunn’s post hoc test). This subtype, along with SD and PLD, presented similar or even slightly greater EM expression than the benign control group, suggesting that the biology of these tumors differs from that of other subtypes.

Considering that FG and PLD are common PC subtypes observed in pathological practice (both can develop from PanIN or IPMN)^5,63^, our results are highly important due to the possible differences in the efficacy of epigenome-targeting therapies. Some of the best-characterized epigenetic-targeting drugs for cancer treatment are HDAC inhibitors (HDACIs), which induce histone hyperacetylation and re-expression of tumor suppressor genes^64^. Until now, the majority of trials on the use of HDACIs for the treatment of PC have not yielded satisfactory results; however, no histological subtyping of patients included in those studies has been performed^35^. This situation might be similar to that of KDM inhibitors^64^. The discrepancies regarding high EZH2 expression and the observed loss of H3K27me3 in PC^50,60,61^ might be explained by the variability in EM expression among the PC subtypes we observed. As EZH2 is known to promote gemcitabine resistance^65,66^, EZH2 inhibition is being evaluated as a potential therapeutic approach. Overall, our findings strongly suggest a variable response to most epigenome-targeting modulators, including HDAC, KDM, or EZH2 inhibitors.

Recent evidence^26^ postulates that the recognition of non-B DNA conformations in cancer and their spatial and relative heterogeneity are equally important to the need to study the genome and epigenome of the disease. Although this is mostly unexplored territory, somewhat because of the lack of suitable technologies, Zhang et al. recently reported that histone acetylation facilitated DNA conformation changes from B-DNA to Z-DNA in HeLa cells^67^. We show that higher acetylation levels (particularly in FG and SD) correlate with a higher Z-DNA ratio. Possible new therapeutic agents, such as those that activate ZBP1, have been shown to sensitize malignant cells to ICB therapy and facilitate necroptotic cell death^26,27^. Here, we report the Z-DNA and B-DNA distributions in PC tissues. We identified PC subtypes (FG and SD) with more regions of DNA in the Z-conformation, suggesting that the PC of FG or SD might be a better candidate for potential ADAR inhibitors or ZBP1 activators to support ICB efficacy.

To conclude, here we propose an innovative approach to spatially examining and semi-quantifying the global distributions of DNA methylation, histone methylation (at lysine and arginine residues), and histone acetylation by multiplexed molecular imaging combined with machine learning and artificial neural networks. Additionally, we suggest the same methodology to be eligible for assessing the levels of Z-DNA conformational changes, which is important information in the context of ADAR inhibitors or ZBP1 activators currently being tested in combination with immune checkpoint blockers. Our proof-of-concept findings revealed significant variability in EMs expression among PC subtypes, demonstrating distinct spatial distributions of this heterogeneity. Specifically, we found that the EM profiles of the FG and SD subtypes differ from those of the other subtypes. Moreover, this study offers a new global perspective on histone arginine methylation in PC. Our results underscore the importance of differentiating PC epigenome studies by histological subtype to enhance the efficacy of therapeutics targeting epigenetic regulators such as HDACs, KDMs, EZH2, ADAR1, or ZBP1. These findings align with current evidence on certain modifications in PC, including reports of global DNA hypomethylation^15,49,50^, histone lysine deacetylation^54–57^, decreased histone lysine methylation^60^, and increased activity of some histone lysine demethylases^58,59^. However, these studies lack important differentiation by PC subtype, a gap our study addresses, potentially explaining the failure of current epigenome-targeting therapies^35^. Although verified spectroscopically, the presented methodological approach is innovative, and thus, further validation studies are required.

## Methods

### Patient selection and tissue sample preparation

Molecular imaging was conducted on 34 PC tissue slides representing 6 histological subtypes: 3 AVAC (containing a total of 32 nuclei), 3 cPDAC (31 nuclei), 8 IPMC (114 nuclei), 4 PLD (53 nuclei), 2 FG (33 nuclei), 2 SD (25 nuclei), and 6 benign CTRL (66 nuclei) specimens. The FG and PLD subtypes were characterized by predominantly (> 50% of the tumor mass) foamy-glands/clear-cell patterns and large-duct/cystic-papillary patterns. The SD samples contained PCs with at least focal squamous differentiation with p40 and p63 immunostaining. IPMC samples contained PCs derived from IPMNs and which invaded mostly as cPDAC, with a single case being PLD. The study included patients who underwent pancreatoduodenectomy (Whipple or Traverso) or distal pancreatectomy for PC diagnosis, excluding those with benign pancreatic or neuroendocrine neoplasms. Tissue samples were obtained from the Pathology Department of Jagiellonian University Medical College and Cracow University Hospital’s archives and were typically stored as formalin-fixed paraffin-embedded (FFPE) blocks. The initial sample selection utilized standard hematoxylin-eosin-stained glass slides (H&E), which were confirmed by two experienced pancreatic pathologists using a routine light microscope. Each chosen case was sliced into a 2.5 μm thick tissue section using a Microm^®^ HM355S Automatic Microtome and mounted onto a CaF_2_ window. Pathologists marked areas of interest on unstained CaF_2_-stained slides, including cancerous cells and stroma. The complete paraffin removal procedure involved a 12-hour xylene bath and graded ethanol rehydration.

### Obtaining optimal lys derivatives for reference spectra

We performed additional processing to optimize the quality of the Raman spectra of Lys, Lys-m, and Lys-a. The final compounds (those best rated by spectrum quality) of Lys, Lys-m, and Lys-a used for acquiring reference spectra were ʟ-lysine hydrochloride, free Nε-methyl-ʟ-lysine, and Nε-acetyl-ʟ-lysine dihydrochloride, respectively.

Unless stated otherwise, all reagents used were of analytical grade and obtained from Sigma Aldrich. Ultrapure water with a resistivity of 18.2 mM was obtained using a Milli-Q water purification system (Merck Millipore).

### Synthesis of ʟ-Lysine hydrochloride (Lys)

A total of 1.46 g (0.01 mol, 1 eq.) of ʟ-lysine was dissolved in 1.67 mL of 6 M HCl (0.01 M, 1 eq.), which was previously cooled to 15 °C in an ice bath. The mixture was then filtered through 0.2 μm Whatman Puradisc 13 syringe filters (Sigma Aldrich) and left to crystallize. The collected crystals were washed three times with 10 mL of anhydrous diethyl ether, left to dry overnight at room temperature, and then dried at 100 °C until no noticeable changes in sample mass were observed.

### Synthesis of Nε-acetyl-ʟ-lysine hydrochloride

Fifty milligrams of Nε-acetyl-ʟ-lysine (0.267 mmol, 1 eq.) was dissolved in 0.265 mL of 0.001 M HCl (0.267 mmol, 1 eq.). The mixture was left to crystallize, and the collected crystals were washed three times with anhydrous diethyl ether and left to dry overnight at room temperature. Then, the sample was dried to a constant mass at 100 °C.

### Synthesis of Nε-acetyl-ʟ-lysine dihydrochloride (Lys-a)

Nε-acetyl-ʟ-lysine dihydrochloride was obtained using the same procedure used for Nε-acetyl-ʟ-lysine hydrochloride, but 0.265 mL of 0.05 M HCl (0.534 mmol, 2 eq.) was used.

### Dehydrochlorination of Nε-methyl-ʟ-lysine hydrochloride (Lys-m)

HCl was removed from Nε-methyl-ʟ-lysine hydrochloride using a modified procedure described by Blomquist et al.^68^. Briefly, 50 mg of Nε-methyl-ʟ-lysine hydrochloride (0.254 mmol, 1 eq.) was dissolved in 0.250 mL of water and cooled in an ice bath to 0 °C. Afterward, 0.250 (1.794 mmol, 7 eq.) mL of ice-cold triethylamine was added, and the obtained mixture was stirred in an ice bath for 30 min. Nε-methyl-ʟ-lysine was precipitated by adding 10 mL of ice-cold acetone. After centrifugation at 30 000 g, the crude product was washed three times with 2 mL of anhydrous chloroform, followed by washing twice with 1 mL of anhydrous diethyl ether. Then, the sample was allowed to dry at room temperature.

### Reference amino acid and DNA conformation measurements

Raman spectra of DNA (Jurkat Genomic DNA, Thermo Fisher Scientific, cat. no SD 1111), methylated DNA (CpG Methylated Jurkat Genomic DNA, Thermo Fisher Scientific, cat. no SD 1121), amino acids and their derivatives were obtained with a Horiba Lab RamHR spectrometer (Horiba Scientific) combined with an upright optical microscope BX41 (Olympus Corporation) equipped with an electron-multiplying charge-coupled device (EM-CCD). Spectra were collected with a green (532 nm) laser, 100× air objective (Olympus Corporation) and 600 mm^− 1^ diffraction grating providing 2 cm^− 1^ spectral resolution. The acquisition time was adjusted for each sample (typically between 10 and 20 s). Measurements were controlled via LabSpec6 software (Horiba Scientific).

To obtain the Raman spectra of the Z-DNA, 10 µL of DNA solution was dissolved in 40 µL of 5 M NaCl (Sigma Aldrich). A 20 µL aliquot of the solution obtained as described above was cast on a microscope slide covered with tin foil. Then, to avoid sample drying, Raman spectra were collected immediately using the procedure described above.

### Raman measurements for RHM plotting

Raman measurements of PC tissue slides were conducted using a Horiba LabRam (Horiba SAS, France) and an Alpha 300R (WITec, Ulm, Germany) confocal Raman microscope system, both of which were equipped with a green (532 nm) laser. The LabRam system was coupled with an electron-multiplying charge-coupled device (EM-CCD) camera cooled to -70 °C. Throughout the measurements, the tissue sections were submerged in a physiological saline solution, and a ×60 water immersion objective lens (Nikon) was utilized. Spectra were captured in the fingerprint spectral range (1900–600 cm^− 1^) with a spectral resolution of 2 cm^− 1^. The RHM technique involves multiple measurements of neighboring “pixels” of tissue, with the resulting spectra combined into a single map image. In this investigation, RHM maps comprised 10,000 to 18,000 spectra for a single slide, with an exposure time of 6 s for each pixel. The size of the pixels (step size) was 1 μm or smaller, depending on the dimensions of the area of interest, ranging from 80 μm × 80 μm to 140 μm × 140 μm.

### High-resolution measurements of selected PC nuclei areas

Confocal Raman spectroscopy measurements were performed using an Alpha 300R confocal Raman microscope system (WITec, Ulm, Germany). The system was equipped with a 532 nm laser light source set to a laser power of 20 mW in front of the objective, a UHTS300 spectrometer with a 600 groove/mm grating and a thermoelectrically cooled (− 60 °C) back-illuminated CCD camera. The spectral data were acquired in an aqueous environment (0.9% NaCl solution) using a 63× water immersion objective NA 1.0 (W Plan-Apochromat, Zeiss, Germany) in the range 100–4100 cm-1. Raman maps of a 100 × 100 µm^2^ scan area (100 × 100 points) were acquired with a continuous laser beam with an accumulation time of 2 s per point. The resolution was improved to 88 × 88 points per 22 × 22 µm^2^ for measurements of the nuclei.

### Spectral preprocessing

Before the CNN can undergo training and be utilized for classification purposes, the RHM spectra must be preprocessed. This involved eliminating signal noise and cosmic ray spikes, as well as normalizing to account for differences in tissue sample thickness or measurement equipment. This preprocessing ensured that the spectral data were comparable, enabling substantive conclusions unaffected by experimental setup variations. In addition to standard preprocessing methods, such as baseline removal (3rd-order polynomial) and cosmic ray spike removal, we employed a novel approach using unsupervised machine learning, specifically AEs. Essentially, an AE comprises two deep artificial neural networks: an encoder, which compresses the data to highlight meaningful features, followed by a decoder, which reconstructs the original data while capturing the extracted features. This spectral data processing method aims to eliminate noise while retaining relevant information. AEs demonstrated high efficiency and accuracy in comparison to the standard approach, as detailed in Supplementary Figure S8 with further description. After denoising, all spectra were normalized in the 0 to 1 range.

### Autoencoder denoising

For efficient spectral preprocessing and smoothing, we utilized a novel machine learning approach, AEs. We used a custom architecture comprising an encoder with 9 convolutional layers and a decoder with 9 convolutional layers; all the layers were finalized with a flat sigmoidal layer. Similar to the classification CNN, a “sequential” base model was employed, and a “glorot uniform” initialization mode was used for each layer. The “Adam” optimizer and “categorical cross-entropy” loss function were utilized. Training with a batch size of 105 was used, and 42 epochs (approx. 10 min) were needed. Details of the proposed AE architecture are summarized in Supplementary Figure S9.

### CNN architecture and training

The CNN was trained to predict three classes representing cellular nuclei, cytoplasm, and surrounding stroma. We employed a custom-designed CNN architecture, as detailed in^15,36^, comprising 10 convolutional layers for feature identification and 4 fully connected layers for classification. The CNN program was executed in Python (RRID: SCR_008394) version 3.10.5 using TensorFlow (RRID: SCR_016345) version 2.9.1 and Keras version 2.9.0. A “sequential” base model was employed, with each layer initialized using the “glorot uniform” mode. The “Adam” optimizer and “categorical cross-entropy” loss function were utilized. The training lasted from 10 to 38 epochs, depending on the RHM map, with a batch size of 105. The spectra for each class were combined and shuffled randomly, followed by “one-hot encoding” of the class arrays. The training and testing datasets were split at a ratio of 70/30. The approximate training time for the CNN was 15 min per RHM map. Further details regarding the CNN architecture are summarized in Supplementary Figure S10.

### CNN-based classification plotting

The CNN-based classification facilitated spectral classification and enabled the detailed identification of PC nuclei. Additionally, the CNN was utilized to generate prediction maps. Each spectrum obtained via RHM for every tissue sample was input into the CNN and categorized into one of three classes. The predicted class values (ranging from 0 to 2 representing stroma/empty, nucleus, and cytoplasm) formed an array, which, when combined with the x and y coordinates of the original RHM map, enabled the creation of a CNN classification map image. Each pixel in the image represents a CNN-predicted class, depicted with a distinct color. These maps were plotted in Python (RRID: SCR_008394) version 3.10.5 with MatPlotLib (RRID: SCR_008624) version 3.5.2.

### Determining the best correlation approach

Before choosing Pearson R for spectral comparison-based analyses, we studied approaches proposed by other authors^46^. Specifically, we evaluated the unit normalized Euclidean distance, squared cosine similarity, squared first-difference cosine similarity, Spearman correlation coefficient, and Pearson correlation coefficient. Then, the correlation spectra of the selected RHM maps were plotted, and the correlation scores were plotted as a heatmap. Visual inspection revealed the best approach for determining the subcellular components of PC tissues. The results of that evaluation are shown in Supplementary Figure S11. All comparisons were custom-coded in Python (RRID: SCR_008394) version 3.10.5.

### Correlation-based analyses and map plotting

Pearson R was selected as the most accurate for spectral matching in complex PC tissue samples. Each spectrum of the RHM map was correlated with the reference spectrum of the EM or DNA conformation, and a score of that correlation was saved with its spatial orientation. Then, all the scores were normalized (between 0 and 1), and heatmaps of the results were generated for subsequent analyses and comparisons. All correlation calculations were custom-coded in Python (RRID: SCR_008394) version 3.10.5.

### Volcano plot-based spectral peak analysis (VSPA)

We utilized a custom approach based on volcano plots to identify spectral features specific to EMs. A volcano plot, when used to compare features, combines the minimal fold change and its statistical significance. We adjusted that idea to reveal statistically significant spectral peaks in the exclusive spectra of EMs: DNA-m, Lys-m/Lys-a, and Arg-m and base-DNA, Lys, and Arg, respectively. Each spectral intensity peak was compared, resulting in a fold change score and its p value (Mann‒Whitney U test). Next, a plot of significant peaks was made by translating volcano plot data to Raman spectrum data, with the log2-fold change value on the y-axis and the Raman shift on the x-axis. The statistically significant (*p* < 0.01) fold changes in the Raman intensities are indicated by red dots. The results of VSPA usage are shown in Supplementary Figures S1-S5.

All calculations and plots were custom-coded in Python (RRID: SCR_008394) version 3.10.5.

### Identification of differentiating spectral features

The identification of spectral features specific to EMs included three steps. The first involved matching the reference spectra of each EM, thus matching DNA-m, Lys-m, Lys-a, and Arg-m with the base spectra and therefore DNA, Lys, and Arg. The manually identified differentiating features are presented in Supplementary Figures S1A – S5A. The next step was to extract exclusive spectra from RHM maps via the process described below (Exclusive spectra extraction subsection). Once found, exclusive EM spectra were compared using two methodologies—PCA and VSPA. The unsupervised machine learning method PCA has been widely used for spectral data comparison, and it has proven to be especially efficient in detecting very slight differences in similar spectra of tissue samples^15,36^. PCA attempts to extract meaningful features from multidimensional data by identifying the direction (called principal components) of the greatest variability in the dataset and by orienting these component variables as orthogonal linear combinations. Here, PCA revealed the grouping of EM vs. base spectra (PCA score count plots in Supplementary Figures S1B and C – S5B and C) and revealed differentiating features of that divergence (PCA loading plots in Supplementary Figures S1C – S5C).

Although PCA is useful for spectral data comparison, it does not provide a statistical estimation of calculations (p values). Consequently, one cannot determine whether the revealed peaks significantly differentiate the studied sample groups. To overcome this issue, we exploited a novel strategy involving the use of so-called volcano plots. Volcano plots are gaining popularity in omics data analyses (i.e., genomics, proteomics, or metabolomics)^69^. This type of statistical plot combines the statistical significance with the so-called fold change of the studied data groups. By adjusting the method used for spectral data analysis, we used volcano plots to identify differentiating spectral peaks among exclusive EM spectra while sustaining the statistical significance and minimal fold-change value.

Spectral features specific to EMs identified in reference spectra were confirmed in both the PCA loading plot and VSPA. PCA, VSPA, and other analyses were custom-coded in Python (RRID: SCR_008394) version 3.10.5 with the SKLearn library (RRID: SCR_019053) version 1.4.1.

### Exclusive spectra extraction

Exclusive spectra compatible with DNA-m, Lys-m, Lys-a, and Arg-m were extracted from those CNNs classified as cancer nuclei and compared to identify spectral differences in certain Raman intensity peaks pointing toward the EM found in the reference data (DNA, Lys, Arg methylation, and Lys acetylation). Strict criteria were applied for the selection of what we called “exclusive spectra”. Specifically, only spectra that were classified as single-EM-positive were left for further analyses (i.e., exclusive DNA-m equals DNA-m-positive, but Lys-m, Lys-a, and Arg-m-negative). This approach allowed for direct spectral comparison and eliminated cumulations of the Raman intensities influenced by multiple modified spectra. The T process of exclusive spectra selection is schematically explained in Fig. 2.

The exclusive spectra classification was performed on the assumption that positively modified spectra should present with the highest correlation with reference samples; thus, the 3rd quartile (0.75) of Pearson R was used as a cutoff point threshold (estimated among CNN-nuclei-only values). This quartile value was a result of arbitrary testing with visual confirmation of the reference DNA correlation (as presented in Fig. 1C). The tested values ranged from 0.50 to 0.95, but 0.75 gave the best results; thus, this value was used for all comparisons and further analyses in the study. Spectral data analyses were custom-coded in Python (RRID: SCR_008394) version 3.10.5.

### Statistical processing

The statistical evaluation of multiple group comparisons involved the Kruskal‒Wallis H test for nonparametric data following Dunn’s post hoc test with p values adjusted using the Holm technique. For the VSPA significance estimation, we used the Mann‒Whitney U test for 2-group comparisons. All tests were performed by custom programming in Python (RRID: SCR_008394) version 3.10.5 using the SciPy library (RRID: SCR_008058) version 1.8.1.

### Ethics approval

The study was conducted following recognized ethical guidelines (Declaration of Helsinki). The project protocol was approved by the Jagiellonian University Bioethics Committee (opinion no. 1072.6120.77.2021).

## Electronic supplementary material

Below is the link to the electronic supplementary material.

Supplementary Material 1

## Data Availability

All data obtained during the studies are available from the corresponding authors upon reasonable request. The custom source code used in this study, along with sample data, is placed in the CodeOcean repository at doi: https://doi.org/10.24433/CO.2120272.v1.

## Abbreviations

PC: Pancreatic cancer
RHM: Raman hyperspectral mapping
EM: Epigenetic modification
PanIN: Pancreatic intraepithelial neoplasia
IPMN: Intraductal papillary mucinous neoplasm
ICB: Immune checkpoint blockade
ADAR1: Adenosine deaminase acting on RNA 1
ZBP1: Z-DNA binding protein 1
HDAC: Histone lysine deacetylase
KDM: Histone lysine demethylase
AE: Autoencoder
CNN: Convolutional neural network
CTRL: Benign pancreatic duct control tissue
cPDAC: Conventional pancreatic ductal adenocarcinoma
IPMC: Adenocarcinoma derived from IPMN
PLD: Predominantly large-duct ductal adenocarcinoma
FG: Foamy-glands/clear-cell ductal adenocarcinoma
SD: Squamous differentiated ductal adenocarcinoma
AVAC: Ampulla of Vater adenocarcinoma
DNA-m: DNA methylation
Lys: lysine
Lys-m: lysine methylation
Lys-a: lysine acetylation
Arg: Arginine
Arg-m: Arginine methylation
PCA: Principal components analysis
VSPA: Volcano plot-based spectral peaks analysis
t-SNE: t-distributed stochastic neighbor embedding
CIN: Chromosomal instability
H3K27me27: Histone trimethylation at Lys27
EZH2: Enhancer of zeste homolog 2

## References

[1] Martens, S. et al. Different shades of pancreatic ductal adenocarcinoma, different paths towards precision therapeutic applications. *Ann. Oncol.* **30**, 1428–1436 (2019). 31161208 10.1093/annonc/mdz181

[2] Collisson, E. A. et al. Subtypes of pancreatic ductal adenocarcinoma and their differing responses to therapy. *Nat. Med.* **17**, 500–503 (2011). 21460848 10.1038/nm.2344 PMC3755490

[3] Moffitt, R. A. et al. Virtual microdissection identifies distinct tumor- and stroma-specific subtypes of pancreatic ductal adenocarcinoma. *Nat. Genet.* **47**, 1168–1178 (2015). 26343385 10.1038/ng.3398 PMC4912058

[4] Bailey, P. et al. Genomic analyses identify molecular subtypes of pancreatic cancer. *Nature* **531**, 47–52 (2016). 26909576 10.1038/nature16965

[5] Szymoński, K., Milian-Ciesielska, K., Lipiec, E. & Adamek, D. Current Pathology Model of Pancreatic Cancer. *Cancers (Basel)*. **14**, 2321 (2022). 35565450 10.3390/cancers14092321 PMC9105915

[6] Reid, M. D. et al. Ampullary carcinoma is often of mixed or hybrid histologic type: an analysis of reproducibility and clinical relevance of classification as pancreatobiliary versus intestinal in 232 cases. *Mod. Pathol.* **29**, 1575–1585 (2016). 27586202 10.1038/modpathol.2016.124

[7] Kim, S. J. et al. Pancreatic ductal adenocarcinoma with a predominant large duct pattern has better recurrence-free survival than conventional pancreatic ductal adenocarcinoma: a comprehensive histopathological, immunohistochemical, and mutational study. *Hum. Pathol.* **127**, 39–49 (2022). 35667635 10.1016/j.humpath.2022.05.018

[8] Martinez-Useros, J., Martin-Galan, M. & Garcia-Foncillas, J. The Match between Molecular subtypes, Histology and Microenvironment of Pancreatic Cancer and its relevance for Chemoresistance. *Cancers (Basel)*. **13**, 322 (2021). 33477288 10.3390/cancers13020322 PMC7829908

[9] Partolina, M. et al. Global histone modification fingerprinting in human cells using epigenetic reverse phase protein array. *Cell. Death Discov*. **3**, 16077 (2017). 28326191 10.1038/cddiscovery.2016.77 PMC5349387

[10] Hofman, P. et al. Current challenges and practical aspects of molecular pathology for non-small cell lung cancers. *Virchows Arch.* **484**, 233–246 (2024). 37801103 10.1007/s00428-023-03651-1 PMC10948551

[11] Saillard, C. et al. Pacpaint: a histology-based deep learning model uncovers the extensive intratumor molecular heterogeneity of pancreatic adenocarcinoma. *Nat. Commun.* **14**, 3459 (2023). 37311751 10.1038/s41467-023-39026-y PMC10264377

[12] Nagtegaal, I. D. et al. The 2019 WHO classification of tumours of the digestive system. *Histopathology* **76**, 182–188 (2020). 31433515 10.1111/his.13975 PMC7003895

[13] Capper, D. et al. DNA methylation-based classification of central nervous system tumours. *Nature* **555**, 469–474 (2018). 29539639 10.1038/nature26000 PMC6093218

[14] Lomberk, G., Dusetti, N., Iovanna, J. & Urrutia, R. Emerging epigenomic landscapes of pancreatic cancer in the era of precision medicine. *Nat. Commun.* **10**, 3875 (2019). 31462645 10.1038/s41467-019-11812-7 PMC6713756

[15] Szymoński, K. et al. Variabilities in global DNA methylation and β-sheet richness establish spectroscopic landscapes among subtypes of pancreatic cancer. *Eur. J. Nucl. Med. Mol. Imaging* ; (2023). 10.1007/s00259-023-06121-7 PMC10119063 36757432

[16] Varier, R. A. & Timmers, H. T. M. Histone lysine methylation and demethylation pathways in cancer. Biochimica et Biophysica Acta (BBA) - reviews on Cancer. ;**1815**:75–89. (2011). 10.1016/j.bbcan.2010.10.002 20951770

[17] Adamek, D. & Stoj, A. Cancer as a mafia within the body: a proposition of conceptual approach that seems congruent to the complex biology of the disease. *Integr. Cancer Sci. Ther.* ;**1**. (2014).

[18] Sherman, M. H. et al. Stromal cues regulate the pancreatic cancer epigenome and metabolome. *Proc. Natl. Acad. Sci.* **114**, 1129–1134 (2017). 28096419 10.1073/pnas.1620164114 PMC5293019

[19] Goymer, P. Natural selection: the evolution of cancer. *Nature* **454**, 1046–1048 (2008). 18756229 10.1038/4541046a

[20] Alexandrov, L. B. et al. The repertoire of mutational signatures in human cancer. *Nature* **578**, 94–101 (2020). 32025018 10.1038/s41586-020-1943-3 PMC7054213

[21] Kim, E., Albrechtsen, N. & Deppert, W. DNA-conformation is an important determinant of sequence-specific DNA binding by tumor suppressor p53. *Oncogene* **15**, 857–859 (1997). 9266973 10.1038/sj.onc.1201412

[22] Herbert, A. Z-DNA and Z-RNA in human disease. *Commun. Biol.* **2**, 7 (2019). 30729177 10.1038/s42003-018-0237-x PMC6323056

[23] Alonso-Curbelo, D. et al. A gene–environment-induced epigenetic program initiates tumorigenesis. *Nature* **590**, 642–648 (2021). 33536616 10.1038/s41586-020-03147-x PMC8482641

[24] Ravichandran, S., Subramani, V. K. & Kim, K. K. Z-DNA in the genome: from structure to disease. *Biophys. Rev.* **11**, 383–387 (2019). 31119604 10.1007/s12551-019-00534-1 PMC6557933

[25] Travers, A. & Muskhelishvili, G. <scp > DNA structure and function</scp >. *FEBS J.* **282**, 2279–2295 (2015). 25903461 10.1111/febs.13307

[26] Zhang, T. et al. ADAR1 masks the cancer immunotherapeutic promise of ZBP1-driven necroptosis. *Nature* **606**, 594–602 (2022). 35614224 10.1038/s41586-022-04753-7 PMC9373927

[27] Meng, L. et al. Mechanisms of immune checkpoint inhibitors: insights into the regulation of circular RNAS involved in cancer hallmarks. *Cell. Death Dis.* **15**, 3 (2024). 38177102 10.1038/s41419-023-06389-5 PMC10766988

[28] Herbert, A. ADAR and Immune silencing in Cancer. *Trends Cancer*. **5**, 272–282 (2019). 31174840 10.1016/j.trecan.2019.03.004

[29] Herbert, A. A genetic instruction code based on DNA conformation. *Trends Genet.* **35**, 887–890 (2019). 31668857 10.1016/j.tig.2019.09.007

[30] Karki, R. & Kanneganti, T-D. ADAR1 and ZBP1 in innate immunity, cell death, and disease. *Trends Immunol.* **44**, 201–216 (2023). 36710220 10.1016/j.it.2023.01.001 PMC9974732

[31] Kuriakose, T. & Kanneganti, T-D. ZBP1: Innate Sensor regulating cell death and inflammation. *Trends Immunol.* **39**, 123–134 (2018). 29236673 10.1016/j.it.2017.11.002 PMC5863909

[32] Hubbard, N. W. et al. ADAR1 mutation causes ZBP1-dependent immunopathology. *Nature* **607**, 769–775 (2022). 35859177 10.1038/s41586-022-04896-7 PMC9339495

[33] Zhang, T. et al. Influenza virus Z-RNAs induce ZBP1-Mediated necroptosis. *Cell* **180**, 1115–1129e13 (2020). 32200799 10.1016/j.cell.2020.02.050 PMC7153753

[34] de Reuver, R. et al. ADAR1 prevents autoinflammation by suppressing spontaneous ZBP1 activation. *Nature* **607**, 784–789 (2022). 35859175 10.1038/s41586-022-04974-w

[35] Hessmann, E., Johnsen, S. A., Siveke, J. T. & Ellenrieder, V. Epigenetic treatment of pancreatic cancer: is there a therapeutic perspective on the horizon? *Gut* **66**, 168–179 (2017). 27811314 10.1136/gutjnl-2016-312539 PMC5256386

[36] Szymoński, K. et al. Combined analytical approach empowers precise spectroscopic interpretation of subcellular components of pancreatic cancer cells. *Anal. Bioanal Chem.* ; (2023). 10.1007/s00216-023-04997-w PMC10684650 37906289

[37] Urbańska, M. et al. Molecular alterations in metaphase chromosomes induced by bleomycin. *Spectrochim Acta Mol. Biomol. Spectrosc.* **312**, 124026 (2024). 10.1016/j.saa.2024.124026 38368817

[38] Luo, X. et al. Plasmonic Gold Nanohole array for surface-enhanced Raman scattering detection of DNA methylation. *ACS Sens.* **4**, 1534–1542 (2019). 31074265 10.1021/acssensors.9b00008

[39] Mello, M. L. S. & Vidal, B. C. Analysis of the DNA fourier transform-infrared microspectroscopic signature using an all-reflecting objective. *Micron* **61**, 49–52 (2014). 24792446 10.1016/j.micron.2014.02.003

[40] Yao, G. & Huang, Q. Theoretical and experimental study of the infrared and Raman spectra of L-lysine acetylation. *Spectrochim Acta Mol. Biomol. Spectrosc.* **278**, 121371 (2022). 10.1016/j.saa.2022.121371 35594700

[41] Xie, Y. et al. In situ exploring Chidamide, a histone deacetylase inhibitor, induces molecular changes of leukemic T-lymphocyte apoptosis using Raman spectroscopy. *Spectrochim Acta Mol. Biomol. Spectrosc.* **241**, 118669 (2020). 10.1016/j.saa.2020.118669 32653824

[42] Fujii, M., Sekine, S. & Sato, T. Decoding the basis of histological variation in human cancer. *Nat. Rev. Cancer*. **24**, 141–158 (2024). 38135758 10.1038/s41568-023-00648-5

[43] Conroy, T. et al. Pancreatic cancer: ESMO clinical practice guideline for diagnosis, treatment and follow-up. *Ann. Oncol.* **34**, 987–1002 (2023). 37678671 10.1016/j.annonc.2023.08.009

[44] Asiala, S. M. & Schultz, Z. D. Surface enhanced Raman correlation spectroscopy of particles in solution. *Anal. Chem.* **86**, 2625–2632 (2014). 24502388 10.1021/ac403882h PMC3966183

[45] Zhang, R. et al. Spectral partition correlation based on Voigt function for Raman spectral library search. *Chemometr. Intell. Lab. Syst.* **215**, 104353 (2021).

[46] Samuel, A. Z. et al. On selecting a suitable spectral matching method for automated analytical applications of Raman spectroscopy. *ACS Omega*. **6**, 2060–2065 (2021). 33521445 10.1021/acsomega.0c05041 PMC7841937

[47] Bararia, A. et al. Differential methylation landscape of pancreatic ductal adenocarcinoma and its precancerous lesions. *Hepatobiliary Pancreat. Dis. Int.* **19**, 205–217 (2020). 32312637 10.1016/j.hbpd.2020.03.010

[48] Choi, I-S. et al. Hypomethylation of LINE-1 and Alu in well-differentiated neuroendocrine tumors (pancreatic endocrine tumors and carcinoid tumors). *Mod. Pathol.* **20**, 802–810 (2007). 17483816 10.1038/modpathol.3800825

[49] Yamamura, K. et al. LINE-1 methylation level and prognosis in pancreas cancer: pyrosequencing technology and literature review. *Surg. Today*. **47**, 1450–1459 (2017). 28536860 10.1007/s00595-017-1539-1

[50] Iguchi, E., Safgren, S. L., Marks, D. L., Olson, R. L. & Fernandez-Zapico, M. E. Pancreatic Cancer, a mis-interpreter of the Epigenetic Language. *Yale J. Biol. Med.* **89**, 575–590 (2016). 28018146 PMC5168833

[51] Jin, B., Robertson, K. D., DNA & Methyltransferases *DNA Damage Repair. Cancer* pp. 3–29. (2013). 10.1007/978-1-4419-9967-2_1 PMC3707278 22956494

[52] Robertson, K. D. DNA methylation and human disease. *Nat. Rev. Genet.* **6**, 597–610 (2005). 16136652 10.1038/nrg1655

[53] Li, J. et al. Non-cell-autonomous cancer progression from chromosomal instability. *Nature* **620**, 1080–1088 (2023). 37612508 10.1038/s41586-023-06464-z PMC10468402

[54] Lomberk, G. et al. Distinct epigenetic landscapes underlie the pathobiology of pancreatic cancer subtypes. *Nat. Commun.* **9**, 1978 (2018). 29773832 10.1038/s41467-018-04383-6 PMC5958058

[55] Schneider, G. et al. Targeting histone deacetylases in pancreatic ductal adenocarcinoma. *J. Cell. Mol. Med.* **14**, 1255–1263 (2010). 19929947 10.1111/j.1582-4934.2009.00974.x PMC3828843

[56] Ouaïssi, M. et al. High histone deacetylase 7 (HDAC7) expression is significantly Associated with adenocarcinomas of the pancreas. *Ann. Surg. Oncol.* **15**, 2318–2328 (2008). 18506539 10.1245/s10434-008-9940-z

[57] Fritsche, P. et al. HDAC2 mediates therapeutic resistance of pancreatic cancer cells via the BH3-only protein NOXA. *Gut* **58**, 1399–1409 (2009). 19528037 10.1136/gut.2009.180711

[58] Hou, X. et al. KDM1A and KDM3A promote tumor growth by upregulating cell cycle-associated genes in pancreatic cancer. *Exp. Biol. Med.* **246**, 1869–1883 (2021). 10.1177/15353702211023473 PMC8424634 34171978

[59] Dandawate, P. et al. The histone demethylase KDM3A, increased in human pancreatic tumors, regulates expression of DCLK1 and promotes tumorigenesis in mice. *Gastroenterology* **157**, 1646–1659e11 (2019). 31442435 10.1053/j.gastro.2019.08.018 PMC6878178

[60] Wei, Y. et al. Loss of trimethylation at lysine 27 of histone H3 is a predictor of poor outcome in breast, ovarian, and pancreatic cancers. *Mol. Carcinog.* **47**, 701–706 (2008). 18176935 10.1002/mc.20413 PMC2580832

[61] Liu, X-Y. et al. Histone methylation in pancreatic cancer and its clinical implications. *World J. Gastroenterol.* **27**, 6004–6024 (2021). 34629816 10.3748/wjg.v27.i36.6004 PMC8476335

[62] Zhang, F. et al. Global analysis of protein arginine methylation. *Cell. Rep. Methods*. **1**, 100016 (2021). 35475236 10.1016/j.crmeth.2021.100016 PMC9017121

[63] Kim, L. et al. Clear cell carcinoma of the pancreas: histopathologic features and a unique biomarker: hepatocyte nuclear factor-1β. *Mod. Pathol.* **21**, 1075–1083 (2008). 18536653 10.1038/modpathol.2008.95

[64] Lomberk, G. A., Iovanna, J. & Urrutia, R. The promise of epigenomic therapeutics in pancreatic cancer. *Epigenomics* **8**, 831–842 (2016). 27337224 10.2217/epi-2015-0016 PMC5066125

[65] Ougolkov, A. V., Bilim, V. N. & Billadeau, D. D. Regulation of pancreatic tumor cell Proliferation and chemoresistance by the histone methyltransferase enhancer of Zeste Homologue 2. *Clin. Cancer Res.* **14**, 6790–6796 (2008). 18980972 10.1158/1078-0432.CCR-08-1013 PMC2690708

[66] Sun, Y. et al. Histone acetyltransferase 1 promotes gemcitabine resistance by regulating the PVT1/EZH2 complex in pancreatic cancer. *Cell. Death Dis.* **12**, 878 (2021). 34564701 10.1038/s41419-021-04118-4 PMC8464605

[67] Zhang, F., Huang, Q., Yan, J. & Chen, Z. Histone acetylation induced transformation of B-DNA to Z-DNA in cells probed through FT-IR spectroscopy. *Anal. Chem.* **88**, 4179–4182 (2016). 27046421 10.1021/acs.analchem.6b00400

[68] Blomquist, A. T., Hiscock, B. F. & Harpp, D. N. A Convenient and effective preparation of amino acids from their Hydrohalide salts. *Synth. Commun.* **3**, 343–346 (1973).

[69] Burger, T. Fudging the volcano-plot without dredging the data. *Nat. Commun.* **15**, 1392 (2024). 38360828 10.1038/s41467-024-45834-7 PMC10869345

