# Supplementary material for: Spatial recognition and semi-quantification of epigenetic events in pancreatic cancer subtypes with multiplexed molecular imaging and machine learning
Source: Sci Rep. 2025 Feb 22;15:6518. doi: 10.1038/s41598-025-90087-z (PMC11846859; doi:10.1038/s41598-025-90087-z)
Supplement: Supplementary file 1 — Supplementary Material 1 [file 41598_2025_90087_MOESM1_ESM.docx]

**Spatial recognition and semi-quantification of epigenetic events in pancreatic cancer subtypes with multiplexed molecular imaging and machine learning**

Krzysztof Szymoński ^1,2*^, Natalia Janiszewska ^3,4^, Kamila Sofińska ^3^, Katarzyna Skirlińska-Nosek ^3,4^, Dawid Lupa ^3^, Michał Czaja ^3,4^, Marta Urbańska ^3,4^, Katarzyna Jurkowska ^3^, Kamila Konik ^5^, Marta Olszewska ^6^, Dariusz Adamek ^1,2^, Kamil Awsiuk ^3^, and Ewelina Lipiec ^3^

^1^ Jagiellonian University, Medical College, Department of Pathomorphology, Cracow, Poland

^2^ Diagnostyka Consilio Sp. z o.o., Cracow, Poland

^3^ Jagiellonian University, Faculty of Physics, Astronomy and Applied Computer Science, M. Smoluchowski Institute of Physics, Cracow, Poland

^4^ Jagiellonian University, Doctoral School of Exact and Natural Sciences, Cracow, Poland

^5^ University Hospital in Cracow, Department of Pathomorphology, Cracow, Poland

^6^ Jagiellonian University, Medical College, Department of Paediatrics, Cracow, Poland

***Corresponding author:** Krzysztof Szymoński,

**Mailing address:** Department of Pathomorphology, Jagiellonian University Medical College, Grzegorzecka 16, 31-531 Cracow, Poland

**E-mail address:** krzysztof.szymonski@uj.edu.pl

**Phone number:** +48 12 421 15 64

**Supplementary Materials**

**
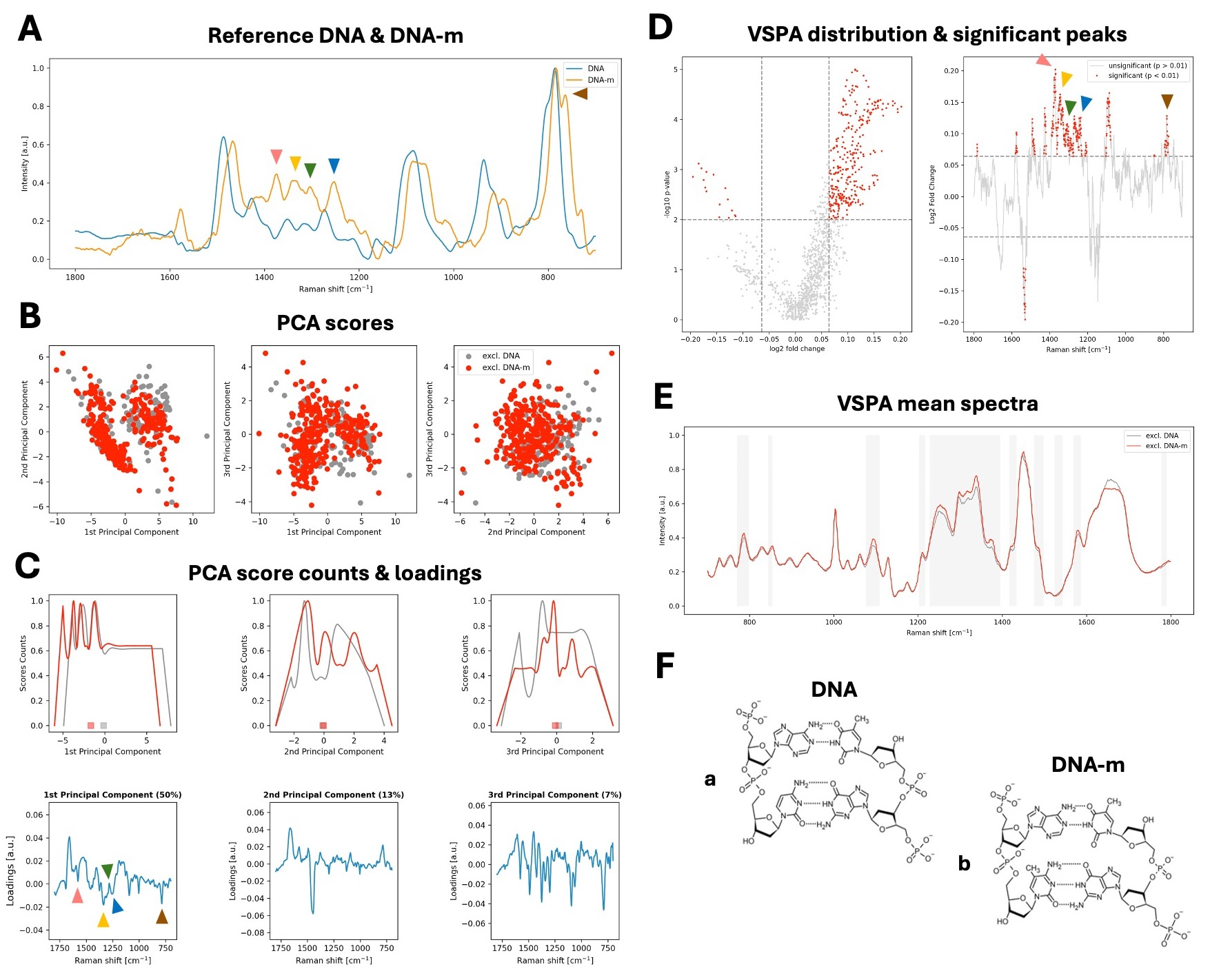
**

**Supplementary Figure S1. The identification of differentiating DNA methylation spectral features in exclusive spectra.** Our approach to confirming DNA-m specificity in spectra extracted with Pearson R correlation-based technique involved three steps: **(A)** the manual recognition of characteristic features in reference spectra of DNA and DNA-m, **(B-C)** PCA of DNA and DNA-m exclusive spectra obtained from PC tissues - here presented with **(B)** 2D scores plots and **(C)** score counts plots. The color squares at the bottom of **(C, top plots)** represent scores median distribution, highlighting the separation between groups, specifically only along 1^st^ principal component. The loadings plots **(C, bottom plots)** identify spectral features of that separation. Next, **(D-E)** VSPA allowed for the identification of significant spectral peaks (p<0.01, Mann–Whitney U test) that manifested with the minimal fold-change (defined as 500 highest fold-change values). Here presented with **(D)** VSPA significant peaks plot and **(E)** VSPA mean spectra plot with certain ranges marked. The same spectral features found in **(A)** were identified in **(C, loadings plot)** and in **(D, significant peaks plot)** – marked by color arrowheads. These are presented in Supplementary Table S1 with the assignment to functional groups vibrations. To illustrate in **(F)** we show the molecular structure of **(F, a)** DNA and **(F, b)** DNA-m. *(DNA-m, DNA methylation; PCA, principal components analysis; VSPA, volcano plot-based spectral peaks analysis)*

**
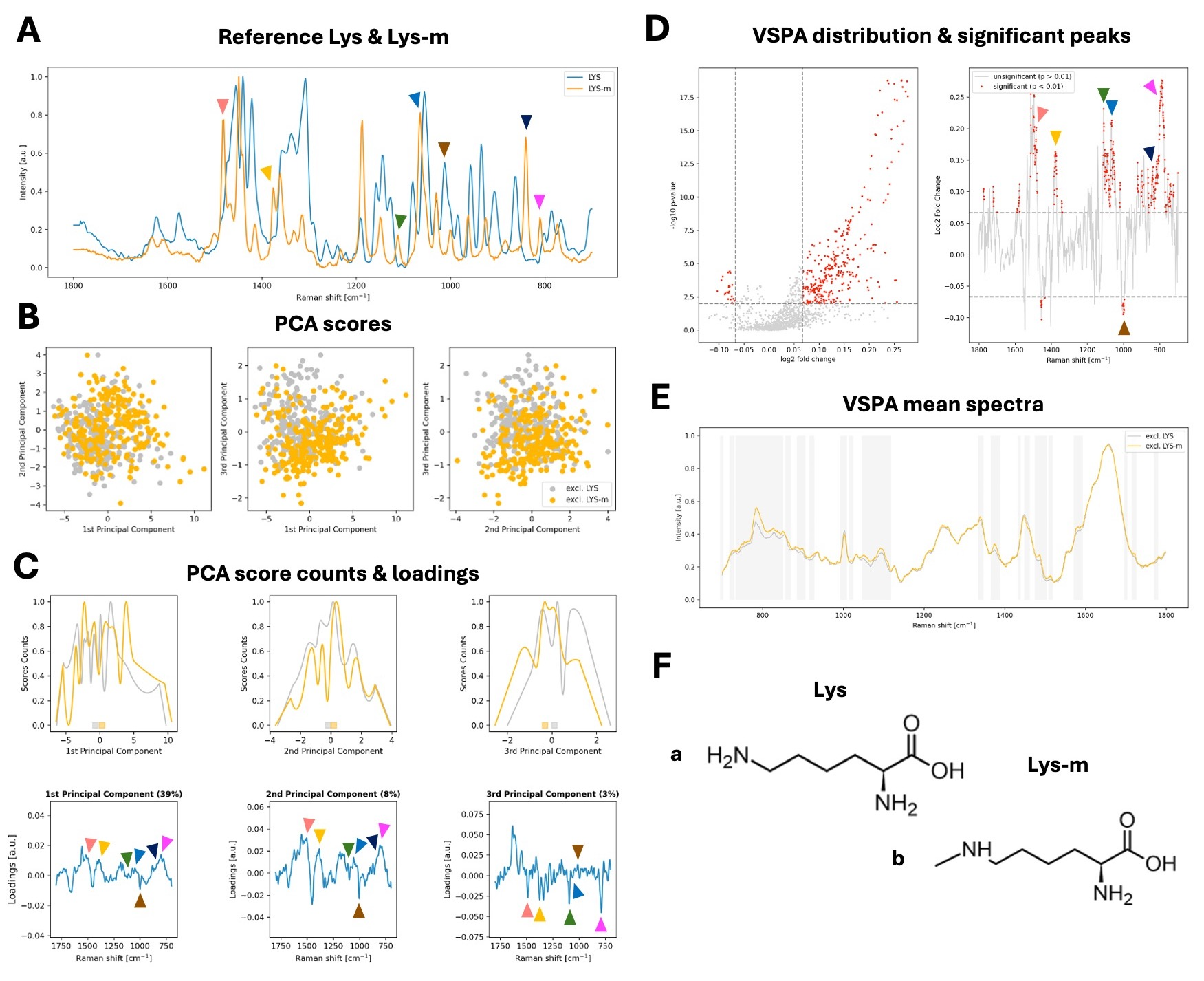
**

**Supplementary Figure S2. The identification of differentiating Lys-m spectral features in exclusive spectra.** Our approach to confirming Lys-m specificity in spectra extracted with Pearson R correlation-based technique involved three steps: **(A)** the manual recognition of characteristic features in reference spectra of Lys and Lys-m, **(B-C)** PCA of Lys and Lys-m exclusive spectra obtained from PC tissues - here presented with **(B)** 2D scores plots and **(C)** score counts plots. The color squares at the bottom of **(C, top plots)** represent scores median distribution, highlighting the separation between groups. The loadings plots **(C, bottom plots)** identify spectral features of that separation. Next, **(D-E)** VSPA allowed for the identification of significant spectral peaks (p<0.01, Mann–Whitney U test) that manifested with the minimal fold-change (defined as 500 highest fold-change values). Here presented with **(D)** VSPA significant peaks plot and **(E)** VSPA mean spectra plot with certain ranges marked. The same spectral features found in **(A)** were identified in **(C, loadings plot)** and in **(D, significant peaks plot)** – marked by color arrowheads. These are presented in Supplementary Table S2 with the assignment to functional groups vibrations. To illustrate in **(F)** we show the molecular structure of **(F, a)** Lys and **(F, b)** Lys-m. *(Lys-m, lysine methylation; PCA, principal components analysis; VSPA, volcano plot-based spectral peaks analysis)*

**
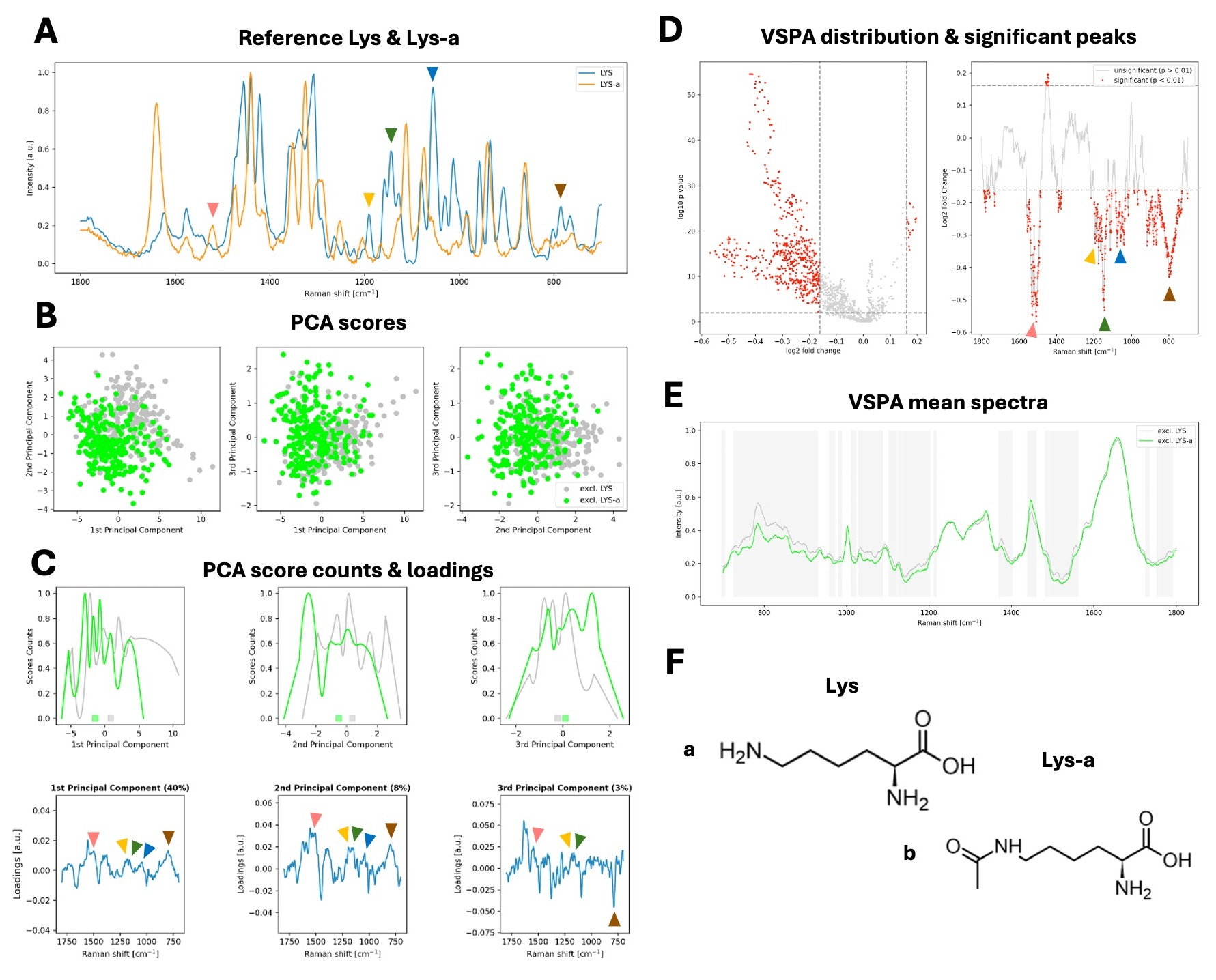
**

**Supplementary Figure S3. The identification of differentiating Lys-a spectral features in exclusive spectra.** Our approach to confirming Lys-a specificity in spectra extracted with Pearson R correlation-based technique involved three steps: **(A)** the manual recognition of characteristic features in reference spectra of Lys and Lys-a, **(B-C)** PCA of Lys and Lys-a exclusive spectra obtained from PC tissues - here presented with **(B)** 2D scores plots and **(C)** score counts plots. The color squares at the bottom of **(C, top plots)** represent scores median distribution, highlighting the separation between groups. The loadings plots **(C, bottom plots)** identify spectral features of that separation. Next, **(D-E)** VSPA allowed for the identification of significant spectral peaks (p<0.01, Mann–Whitney U test) that manifested with the minimal fold-change (defined as 500 highest fold-change values). Here presented with **(D)** VSPA significant peaks plot and **(E)** VSPA mean spectra plot with certain ranges marked. The same spectral features found in **(A)** were identified in **(C, loadings plot)** and in **(D, significant peaks plot)** – marked by color arrowheads. These are presented in Supplementary Table S3 with the assignment to functional groups vibrations. To illustrate in **(F)** we show the molecular structure of **(F, a)** Lys and **(F, b)** Lys-a. *(Lys-a, lysine acetylation; PCA, principal components analysis; VSPA, volcano plot-based spectral peaks analysis)*

**
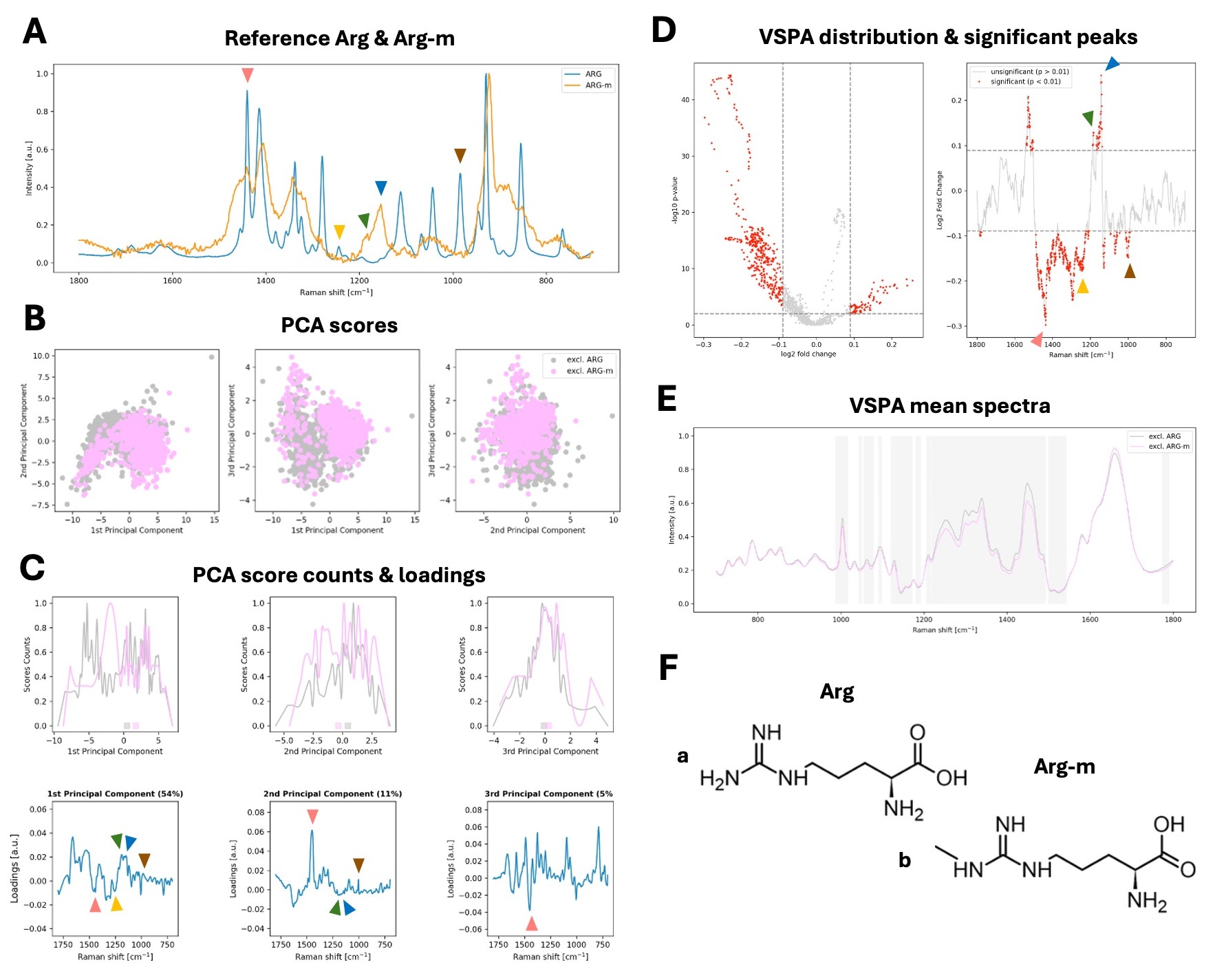
**

**Supplementary Figure S4. The identification of differentiating Arg-m spectral features in exclusive spectra.** Our approach to confirming Arg-m specificity in spectra extracted with Pearson R correlation-based technique involved three steps: **(A)** the manual recognition of characteristic features in reference spectra of Arg and Arg-m, **(B-C)** PCA of Arg and Arg-m exclusive spectra obtained from PC tissues - here presented with **(B)** 2D scores plots and **(C)** score counts plots. The color squares at the bottom of **(C, top plots)** represent scores median distribution, highlighting the separation between groups. The loadings plots **(C, bottom plots)** identify spectral features of that separation. Next, **(D-E)** VSPA allowed for the identification of significant spectral peaks (p<0.01, Mann–Whitney U test) that manifested with the minimal fold-change (defined as 500 highest fold-change values). Here presented with **(D)** VSPA significant peaks plot and **(E)** VSPA mean spectra plot with certain ranges marked. The same spectral features found in **(A)** were identified in **(C, loadings plot)** and in **(D, significant peaks plot)** – marked by color arrowheads. These are presented in Supplementary Table S4 with the assignment to functional groups vibrations. To illustrate in **(F)** we show the molecular structure of **(F, a)** Arg and **(F, b)** Arg-m. *(Arg-m, arginine methylation; PCA, principal components analysis; VSPA, volcano plot-based spectral peaks analysis)*

**
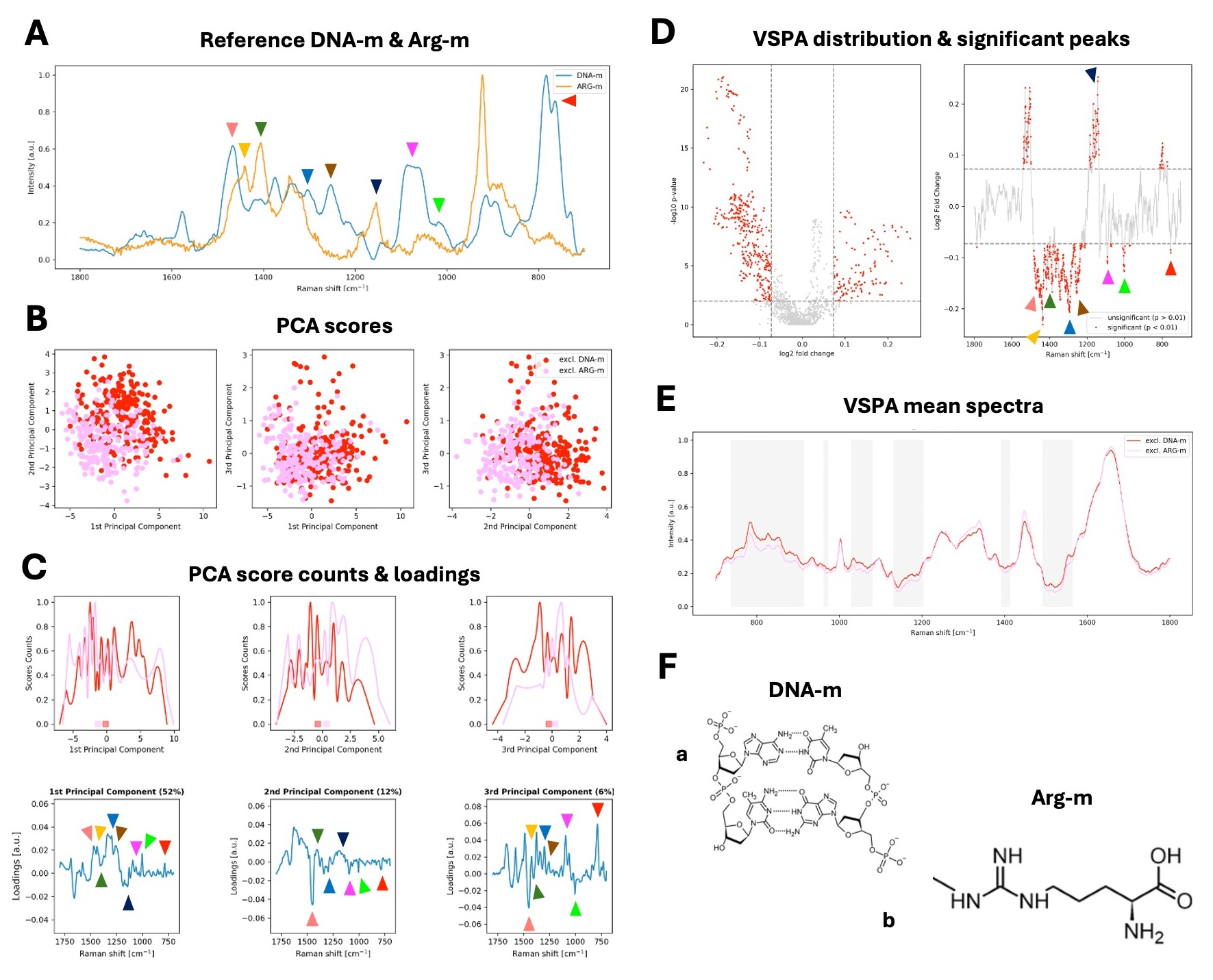
**

**Supplementary Figure S5. The identification of spectral features differentiating DNA-m and Arg-m in exclusive spectra.** Our approach to confirming DNA-m and Arg-m specificity in spectra extracted with Pearson R correlation-based technique involved three steps: **(A)** the manual recognition of characteristic features in reference spectra of DNA-m and Arg-m, **(B-C)** PCA of DNA-m and Arg-m exclusive spectra obtained from PC tissues - here presented with **(B)** 2D scores plots and **(C)** score counts plots. The color squares at the bottom of **(C, top plots)** represent scores median distribution, highlighting the separation between groups. The loadings plots **(C, bottom plots)** identify spectral features of that separation. Next, **(D-E)** VSPA allowed for the identification of significant spectral peaks (p<0.01, Mann–Whitney U test) that manifested with the minimal fold-change (defined as 500 highest fold-change values). Here presented with **(D)** VSPA significant peaks plot and **(E)** VSPA mean spectra plot with certain ranges marked. The same spectral features found in **(A)** were identified in **(C, loadings plot)** and in **(D, significant peaks plot)** – marked by color arrowheads. These are presented in Supplementary Table S5 with the assignment to functional groups vibrations. To illustrate in **(F)** we show the molecular structure of **(F, a)** DNA-m and **(F, b)** Arg-m. *(DNA-m, DNA methylation; Arg-m, arginine methylation; PCA, principal components analysis; VSPA, volcano plot-based spectral peaks analysis)*

**Supplementary Table S1. Assignment of Raman bands identified as specific for DNA and DNA methylation with the analytical approach presented in Supplementary Figure S1.**

| Raman shift [cm^-1^] | Functional groups vibrations* | Reference |
| --- | --- | --- |
| 1375 (m) | A, T, G | ^1^ |
| 1340 (m) | A | ^2^ |
| 1304 (m) | A, T, G, C | ^3^ |
| 1252 (m) | G, C | ^3^ |
| 765 (m) | DNA bkb | ^4^ |

** A, adenine; T, thymine; C, cytosine; G, guanine; bkb, phosphate backbone*

**Supplementary Table S2. Assignment of Raman bands identified as specific for lysine and lysine methylation with the analytical approach presented in Supplementary Figure S2.**

| Raman shift [cm^-1^] | Functional groups vibrations* | Reference |
| --- | --- | --- |
| 1485(m) | δ NH_3_^+^ | ^5^ |
| 1375(m) | δ CH_2_ | ^5^ |
| 1110(m) | τ NH_2_ | ^5^ |
| 1064(m) | *v* CN, *v* CC | ^5^ |
| 1012 | *v* CN, *v* CC | ^5^ |
| 840(m) | δ COO^-^ | ^5^ |
| 808(m) | δ COO^-^ | ^5^ |

* *ν*, stretching; δ, bending; τ, twisting; ρ, rocking; ω, wagging

**Supplementary Table S3. Assignment of Raman bands identified as specific for lysine and lysine acetylation with the analytical approach presented in Supplementary Figure S3.**

| Raman shift [cm^-1^] | Functional groups vibrations* | Reference |
| --- | --- | --- |
| 1521(a) | *ν* NH_3_^+^ | ^6^ |
| 1190 | τ NH_2_ | ^6^ |
| 1145 | τ NH_2_ | ^6^ |
| 1055 | *v* CN, *v* CC | ^6^ |
| 785 | δ COO^-^ | ^6^ |

* *ν*, stretching; δ, bending; τ, twisting; ρ, rocking; ω, wagging

**Supplementary Table S4. Assignment of Raman bands identified as specific for arginine and arginine methylation with the analytical approach presented in Supplementary Figure S4.**

| Raman shift [cm^-1^] | Functional groups vibrations* | Reference |
| --- | --- | --- |
| 1440 | δ CH_2_ | ^1^ |
| 1243 | δ CH_2_ | ^1^ |
| 1183(m) | ρ NH_3_^+^ | ^1^ |
| 1154(m) | δ_asym_ C-N_2_-H_2_ | ^7^ |
| 984 | ρ CH_2_ | ^1^ |

* *ν*, stretching; δ, bending; δ_asym_, asymmetric bending; τ, twisting; ρ, rocking; ω, wagging

**Supplementary Table S5. Assignment of Raman bands identified as specific for Arginine and Arginine methylation with the analytical approach presented in Supplementary Figure S5.**

| Raman shift [cm^-1^] | Functional groups vibrations* | Reference |
| --- | --- | --- |
| 1467 | δ CH_2_, CH_3_ | ^1^ |
| 1441 | δ CH_2_ | ^6^ |
| 1406 | *ν* COO^-^ | ^6^ |
| 1304 | A, T, C, G | ^3^ |
| 1252 | G, C | ^3^ |
| 1154 | δ_asym_ C-N_2_-H_2_ | ^7^ |
| 1086 | *ν* PO_2_^-^ | ^8^ |
| 1076 | *ν* CN | ^6^ |
| 1018 | ρ CH_3_ | ^9^ |
| 764 | *ν* OPO | ^10^ |

* *ν*, stretching; δ, bending; δ_asym_, asymmetric bending; τ, twisting; ρ, rocking; ω, wagging; *A, adenine; T, thymine; C, cytosine; G, guanin*


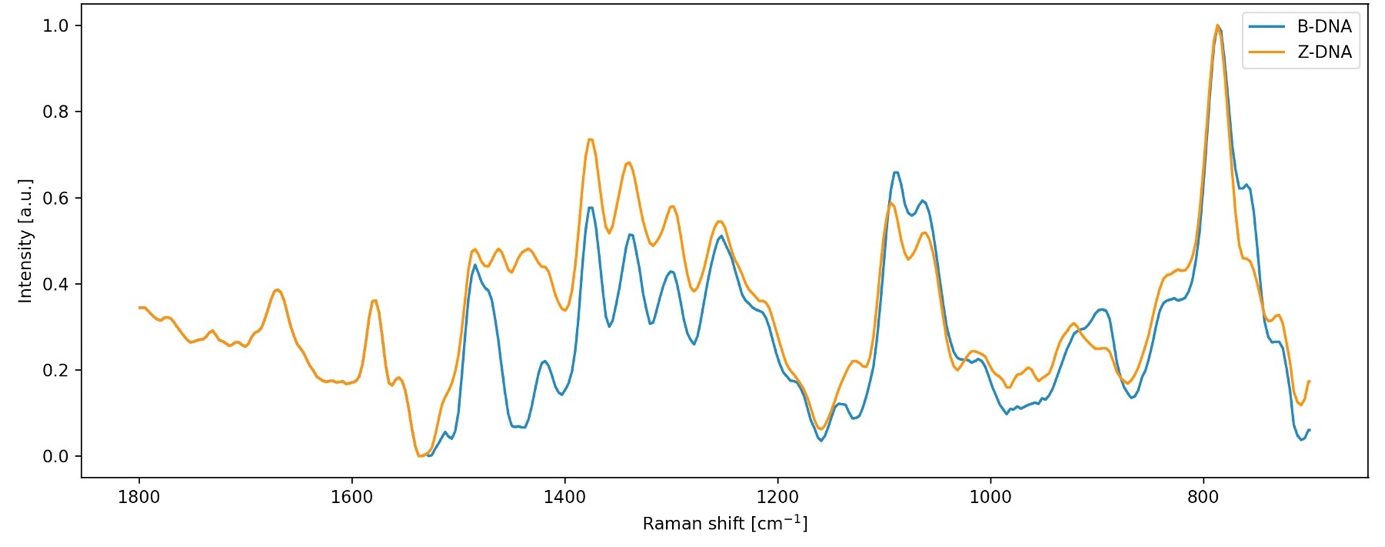


**Supplementary Figure S6. Reference spectra of DNA in right-handed B-DNA and left-handed Z-DNA conformations.** These spectra were used as a reference for correlation-based assessment of DNA conformations in PC tissues.


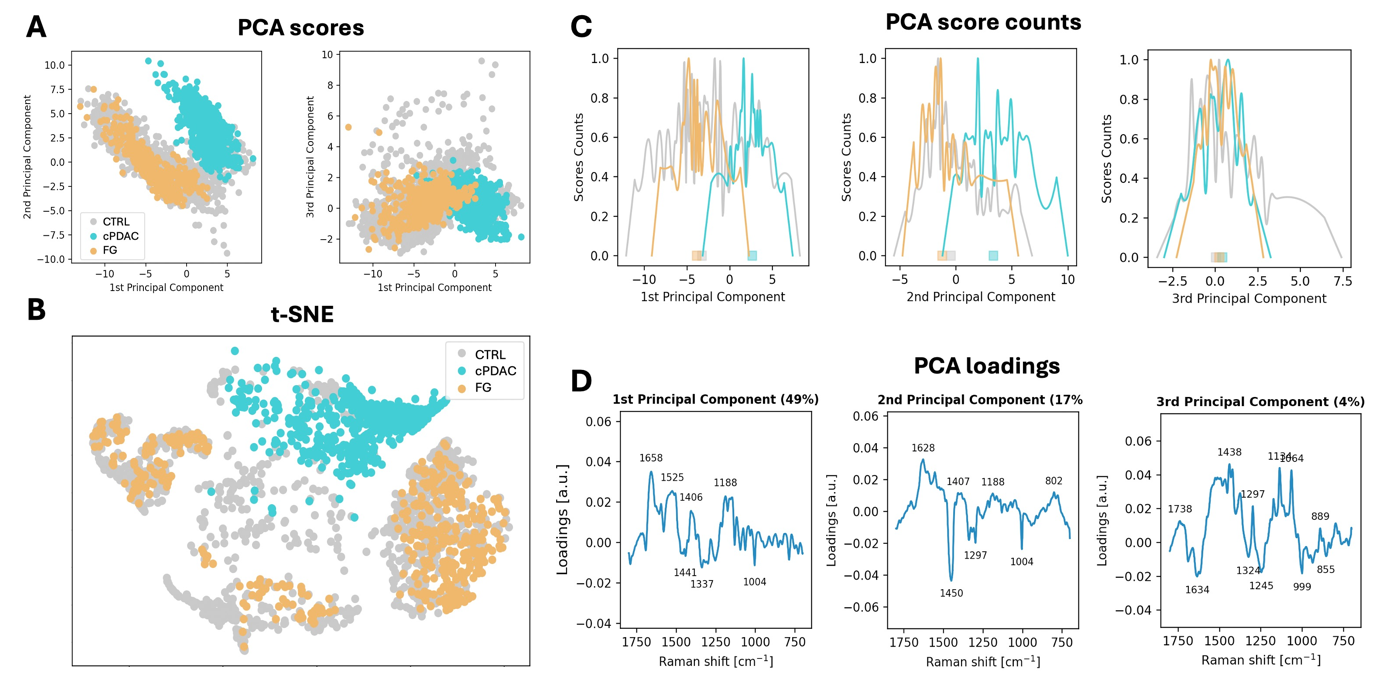


**Supplementary Figure S7. The results of PCA and t-SNE analysis performed on chromatin spectra acquired from CTRL, cPDAC, and FG tissue samples.** PCA scores plots **(A)**, t-SNE results **(B)**, and PCA score counts **(C)** visualize the clustering of similar spectra and the separation between spectra of various PC subtypes. The PCA loadings **(D)** highlight Raman bands from amides, and methyl and methylene motions driving the separation, including: i) the positive correlation of the PC-1 loading with the Amide I at 1658 cm^-1^, indicating the relatively high content of α-helical proteins (cPDAC and partially CTRL); ii) methyl and methylene deformational motions at 1441cm^-1^ and 1337cm^-1^ and methyl deformation of cytosine at 1406 cm^-1^ ^11^, which confirms differences in DNA methylation level in the investigated tissues; iii) the asymmetric bending (O–P–O) of RNA and C3’ endo-sugar phosphate from DNA at 1188 cm^-1^, related to DNA conformational transition; iv) the positive correlation of the PC-2 loading with the Amide I at 1628 cm^-1^, indicating the relatively high content of β-sheet secondary structure in proteins of CPDAC in comparison with FG and CTRL.

The abovementioned bands are responsible for the clustering of spectra presented in Figure 6 of the manuscript, however, for the sake of clarity and transparency, here, the interpretation of chemometric analysis was performed for this limited dataset. *(PCA, principal components analysis; PC-1 and 2, principal components 1 and 2t-SNE, t-distributed stochastic neighbor embedding; CTRL, benign control pancreatic duct samples; cPDAC, conventional ductal adenocarcinoma; FG, foamy-glands ductal adenocarcinoma)*


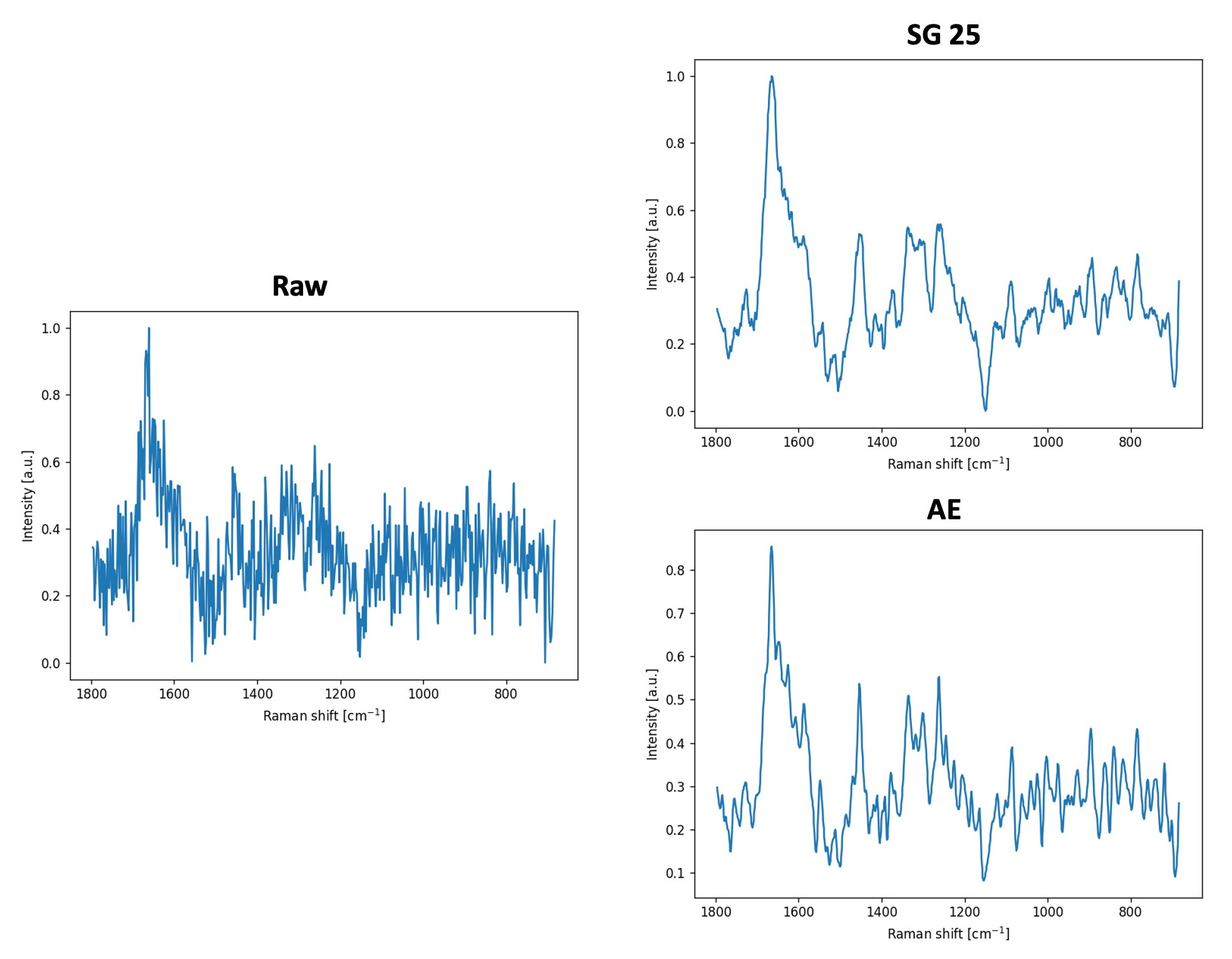


**Supplementary Figure S8. Comparison of standard and machine learning-based approach to spectra pre-processing.** Autoencoder is a machine learning technique that is based on two artificial neural networks, specifically convolutional neural networks. First, the *encoder*, extracts the most important features of a dataset, by limiting its dimensionality. Then, the process is reversed, by the *decoder*, which restores the initial data from the encoder-extracted features list. The process is automatic, flexible, and efficient for large datasets. Here as an example, a comparison of the Savitzky-Golay standard approach **(SG 25)** and the result of autoencoder **(AE)** processing is shown. The AE seems to better highlight meaningful features compared to SG 25.


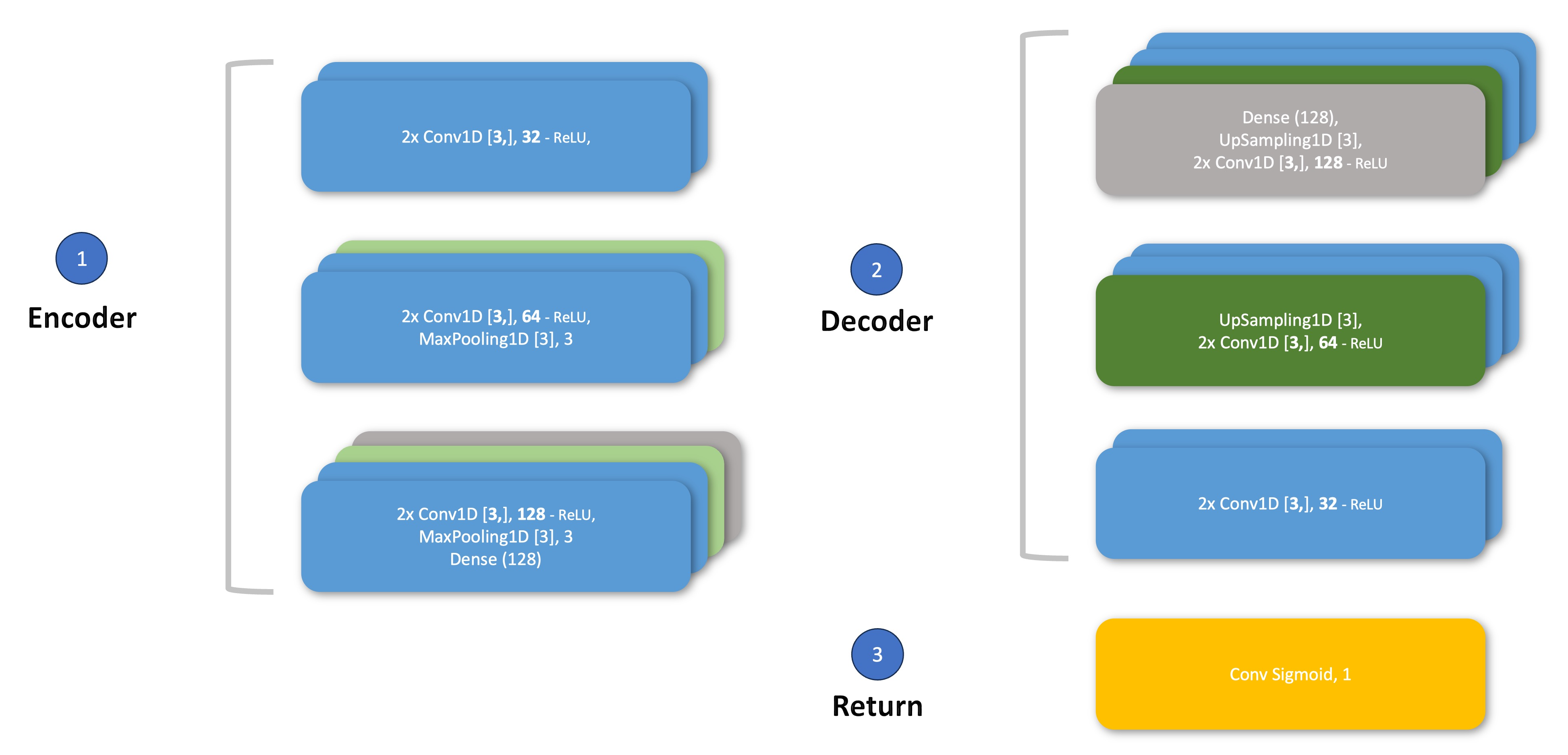


**Supplementary Figure S9. The autoencoder architecture used in the study.** We exploited a custom-designed autoencoder architecture. Specifically, the encoder **(1)** comprised a block of 2 convolutional layers with 32 filters (kernel size [3,], ReLu activation), followed by another block of 2 convolutional layers with 64 filters (same setting) and a max pooling layer (pool size 3, stride 3), and final block of of 2 convolutional layers with 128 filters (same setting), a max pooling layer (same setting), and a dense layer with 128 neurons. The decoder **(2)** reversed the encoder and finalized with a sigmoid return **(3)** layer.


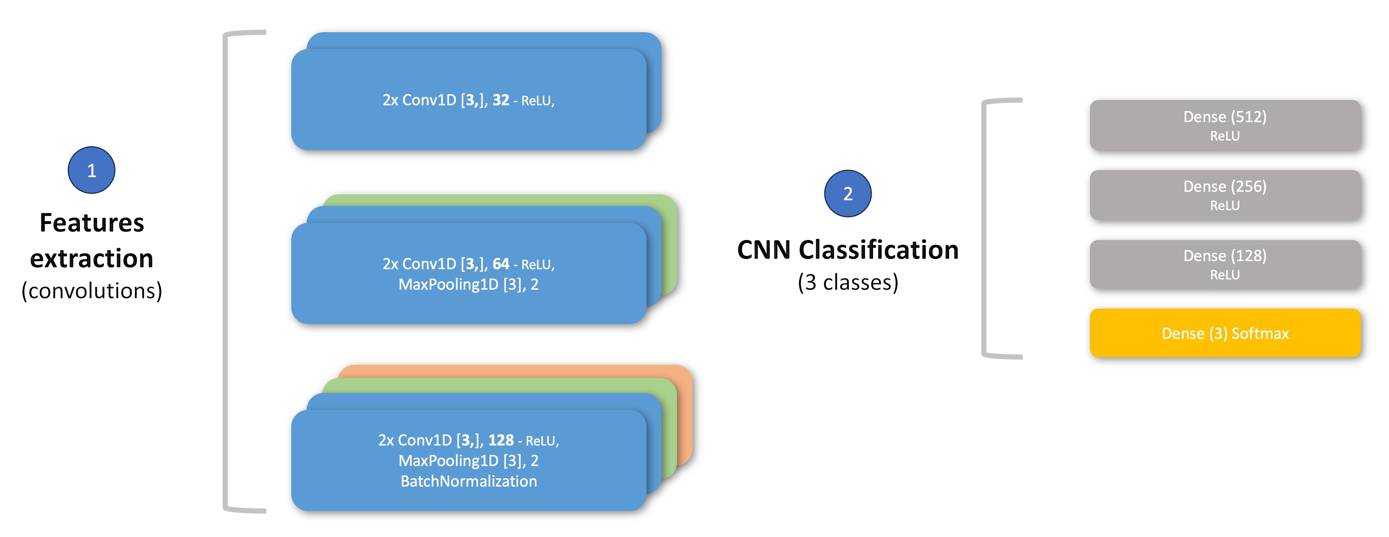


**Supplementary Figure S10. The architecture of convolutional neural network used for spectra classification.** A custom-designed CNN architecture was used for spectra classification into 3 classes, specifically the nuclei, cytoplasm, and tumor stroma. First features extraction **(1)** was utilized by 3 sets of convolutional layers: 2x with 32 filters, then 2x 64 filters and a max pooling layer, and 2x 128 filters, max pooling, and a batch normalization layer. All convolutional layers comprised of kernel size [3,] and were ReLu-activated. After features extraction for the process was finalized with a fully connected set of classification layers **(2)**, specifically of 512, 256, 128 neurons, and the resulting 3 neurons (Softmax).


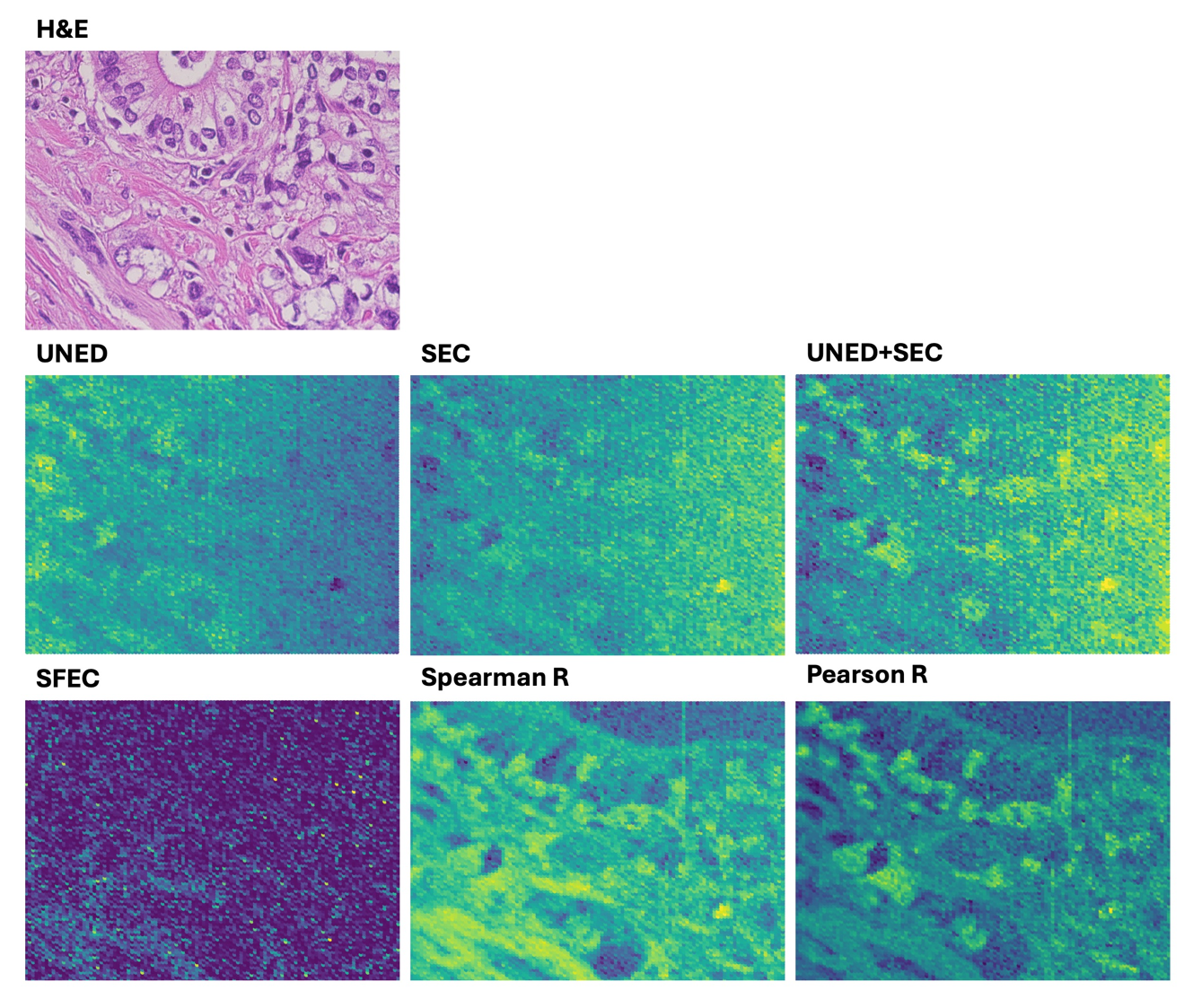


**Supplementary Figure S11. A comparison study of best correlation-based approach technique.** Various methods of spectral correlation were investigated, and judged on the best projection of PC tissues, based on the hematoxilin-&-eosin-stained slide **(H&E)**. Specifically, unit normalized Euclidean distance **(UNED)**, squared cosine similarity **(SEC)**, UNED and SEC combined **(UNED+SEC)**, squared first-difference cosine similarity **(SFEC)**, Spearman correlation coefficient **(Spearman R)**, and Pearson correlation coefficient **(Pearson R)** were assessed. Spearman R and Pearson R delivered similar results, although Pearson R was slightly more accurate in reproducing subcellular components (i.e. cancer cells nuclei).

**References:**

1. Gorelik, V. S., Krylov, A. S. & Sverbil, V. P. Local Raman spectroscopy of DNA. *Bulletin of the Lebedev Physics Institute* **41**, 310–315 (2014).
2. Prescott, B., Steinmetz, W. & Thomas, G. J. Characterization of DNA structures by laser Raman spectroscopy. *Biopolymers* **23**, 235–256 (1984).
3. Movasaghi, Z., Rehman, S. & Rehman, I. U. Raman Spectroscopy of Biological Tissues. *Appl Spectrosc Rev* **42**, 493–541 (2007).
4. Peticolas, W. L., Patapoff, T. W., Thomas, G. A., Postlewait, J. & Powell, J. W. Laser Raman Microscopy of Chromosomes in Living Eukaryotic Cells: DNA Polymorphism In Vivo. *Journal of Raman Spectroscopy* **27**, 571–578 (1996).
5. Aliaga, A. E. *et al.* Surface enhanced Raman scattering study of l-lysine. *Vib Spectrosc* **50**, 131–135 (2009).
6. Aliaga, A. E. *et al.* SERS and theoretical studies of arginine. *Spectrochim Acta A Mol Biomol Spectrosc* **76**, 458–463 (2010).
7. Bhunia, S., Srivastava, S. K., Materny, A. & Ojha, A. K. A vibrational and conformational characterization of arginine at different pH values investigated using Raman spectroscopy combined with DFT calculations. *Journal of Raman Spectroscopy* **47**, 1073–1085 (2016).
8. Samuel, A. Z., Sugiyama, K., Ando, M. & Takeyama, H. Direct imaging of intracellular RNA, DNA, and liquid–liquid phase separated membraneless organelles with Raman microspectroscopy. *Commun Biol* **5**, 1383 (2022).
9. Li, L., Lim, S. F., Puretzky, A., Riehn, R. & Hallen, H. D. DNA Methylation Detection Using Resonance and Nanobowtie-Antenna-Enhanced Raman Spectroscopy. *Biophys J* **114**, 2498–2506 (2018).
10. Thomas, G. J. *et al.* Polarized Raman spectra of oriented fibers of A DNA and B DNA: anisotropic and isotropic local Raman tensors of base and backbone vibrations. *Biophys J* **68**, 1073–1088 (1995).
11. Lipiec, E. *et al.* Infrared nanospectroscopic mapping of a single metaphase chromosome. *Nucleic Acids Res* **47**, e108–e108 (2019).
